# Structural and Functional Treatment of Persistent Spinal Pain Syndrome Type 2: A Fibrosis-Stratified Network Meta-Analysis of Epidural Adhesiolysis and Neuromodulation

**DOI:** 10.3390/jcm15166480

**Published:** 2026-08-21

**Authors:** Wolfgang Auffermann, Mohammed Al Jumaily, Alina Auffermann

**Affiliations:** 1 Department of Interventional Radiology, Dr. Sulaiman Al Habib Hospital, Dubai 505005, United Arab Emirates; 2 Department of Biomedical Engineering, Hamburg University of Applied Sciences (HAW Hamburg), 21033 Hamburg, Germany; 3 American Spine Center, Dubai P.O. Box 99227, United Arab Emirates; 4 Department of Health and Applied Social Sciences, John Moores University, Liverpool L3 5AH, UK; 5 German Clinic, Al Razi Medical Complex, Dubai Healthcare City, Dubai 505005, United Arab Emirates

**Keywords:** fibrosis, post-laminectomy syndrome, failed back surgery syndrome, persistent spinal pain, adhesiolysis, spinal cord stimulation

## Abstract

**Background/Objectives**: Persistent spinal pain syndrome type 2 (PSPS-T2) is treated with two mechanistically distinct interventional families: epidural adhesiolysis (EA), directed at structural epidural pathology, and neuromodulation (NM), directed at modulation of nociceptive signaling. Epidural fibrosis is a biologically plausible candidate for treatment stratification, but whether it independently modifies treatment response remains unproven. This study reviewed the available evidence to determine both the comparative findings and the limitations imposed by the current evidence architecture. **Methods**: A PROSPERO-registered systematic review and frequentist random-effects network meta-analysis were performed. Studies were classified at the study level, rather than the individual-patient level, as fibrosis-positive (structurally confirmed) or unselected (without confirmed fibrosis). Treatment effects were synthesized separately within each stratum, and an exploratory study-level meta-regression evaluated fibrosis status as a potential moderator. **Results:** The final dataset comprised 87 studies including 33,504 patients: 40 fibrosis-positive studies (n = 4180) and 47 fibrosis-unselected studies (n = 29,324). Treatment family and fibrosis status were almost completely confounded. EA was evaluated almost exclusively in fibrosis-positive cohorts, whereas NM was evaluated almost exclusively in fibrosis-unselected studies. Although pooled improvements in pain (−0.97 vs. −0.63) and disability (−11.01 vs. −8.46) were greater in fibrosis-positive studies, these differences cannot be attributed independently to fibrosis. Residual heterogeneity remained substantial (I^2^ 71–92%). Peripheral nerve field stimulation and EA protocols incorporating steroid, hyaluronidase, and hypertonic saline achieved the highest probability score for pain relief. All probability scores for the treatments should be interpreted as exploratory. **Conclusions:** Discordance between EA and NM in PSPS-T2 stems from structurally different populations, precluding pooled comparative classifications or claims that fibrosis modifies treatment effect. While fibrosis remains a promising candidate stratifier, proving it requires prospective trials that stratify randomization to EA versus NM following standardized fibrosis assessment.

## 1. Introduction

### 1.1. The Clinical Problem

Persistent spinal pain syndrome type 2 (PSPS-T2), formerly failed back surgery syndrome (FBSS), affects 20–40% of patients after lumbar spine surgery [1,2,3]. Despite technically successful procedures, these patients experience disabling back and leg pain, leading to opioid dependence, unemployment, and profound quality-of-life impairment [4,5,6]. Two interventional treatments have emerged: epidural adhesiolysis (EA) and neuromodulation (NM) [4,7,8]. EA and NM are characterized by fundamentally distinct mechanisms of action. EA is conceptually aligned with structural pathology because it aims to disrupt adhesions, improve target-site access, and restore nerve-root mobility. NM, in contrast, is aligned with functional modulation of nociceptive signaling and network-level pain processing. Yet, despite decades of clinical use, no evidence-based framework exists to guide treatment selection. This gap has led to conflicting practice patterns and suboptimal outcomes for many patients. Spinal disorders and chronic pain syndromes together constitute one of the largest contributors to years lived with disability in high-income settings, and the rehabilitation evidence base for these conditions has been systematically catalogued, underlining both the scale of the burden and the limitations of current treatment evidence [9,10].

### 1.2. Epidural Fibrosis as a Biologically Active Structural Lesion

Among the myriad etiologies of PSPS-T2, epidural fibrosis—scar tissue formation in the epidural space following laminectomy—represents a common yet frequently overlooked mechanism [11,12]. Ross and colleagues [13] demonstrated that patients with extensive epidural scarring are 3.2 times more likely to experience recurrent radicular pain. In symptomatic FBSS patients undergoing epiduroscopy, significant epidural fibrosis was identified in 91.0% and severe fibrosis in 83.3%, whereas magnetic resonance imaging (MRI) detected fibrosis in only 16.1%, underscoring that clinically relevant postoperative fibrosis may be substantially underrecognized when ascertainment relies on conventional imaging alone [12].

Epidural fibrosis is not merely mechanical scarring, but a dynamic biological process driven by dysregulated wound healing [11,14,15,16]. Following laminectomy, the disruption of normal epidural tissue planes initiates a cascade of inflammatory and fibrotic responses. Within hours, platelet degranulation releases transforming growth factor-β1 (TGF-β1), a central regulator of fibrosis, which stimulates fibroblast proliferation and differentiation into myofibroblasts [15,16]. These myofibroblasts deposit excessive extracellular matrix components including collagen type I and III, fibronectin, and hyaluronic acid [17,18].

The fibrotic response is further shaped by imbalance between matrix metalloproteinases (MMPs) and their inhibitors (TIMPs), favoring extracellular matrix accumulation over degradation [19,20]. Preclinical post-laminectomy data further support that epidural fibrosis is a biologically active process rather than inert scar tissue [21]. The resulting scar tethers nerve roots, restricts their normal mobility, compromises microcirculation, and traps inflammatory mediators including interleukin-1β (IL-1β) and tumor necrosis factor-α (TNF-α) [22,23,24]; how far these findings translate to human postoperative fibrosis has not been established directly.

**Figure 1:**
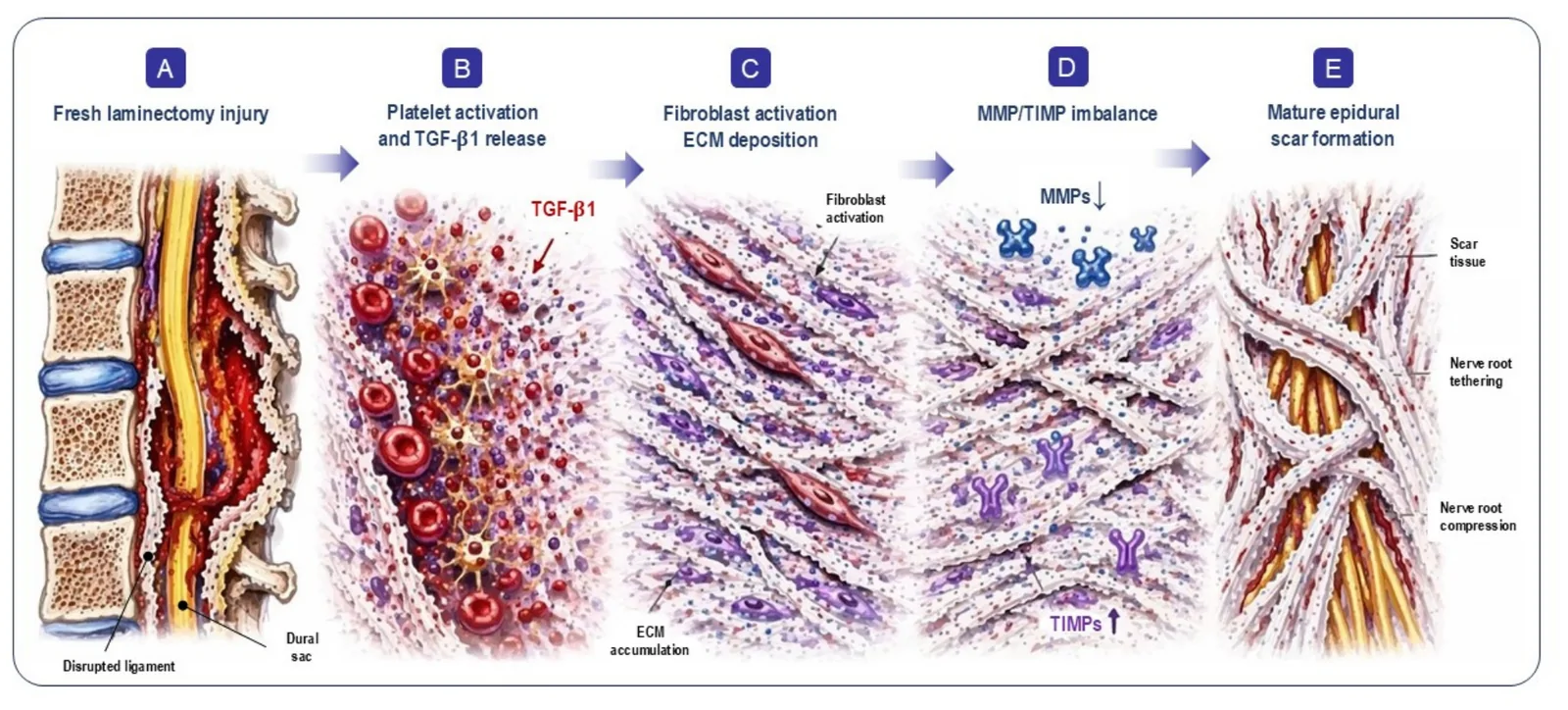
**Preclinical mechanism of molecular pathogenesis of epidural fibrosis. Schematic illustration of the molecular cascade following lumbar laminectomy**. (**A**) Surgical injury disrupts posterior stabilizing structures and epidural fat, (**B**) triggering platelet degranulation and release of transforming growth factor-β1 (TGF-β1). (**C**) TGF-β1 activates fibroblasts and promotes differentiation into matrix-producing myofibroblasts, leading to excessive extracellular matrix deposition (ECM). (**D**) Dysregulation of matrix metalloproteinases (MMPs) and their inhibitors (TIMPs) shifts the balance toward matrix accumulation and impaired remodeling. (**E**) Progressive scar organization culminates in mature epidural fibrosis with nerve root tethering and compression [11,14,15,16,17,18,19,20,21,22,23,24]. ↓ decrease; ↑ increase. Figure created by the authors.

This proposed cascade—from surgical injury through TGF-β1 activation and matrix accumulation to mature scar—positions epidural fibrosis as a biologically active structural lesion rather than an inert radiological finding, and provides the rationale for examining it as a candidate stratifier of treatment response. It is a mechanistic rationale drawn largely from preclinical work, and is presented here as motivation for the question addressed rather than as established human pathophysiology (Figure 1).

### 1.3. Studies Without Confirmed Fibrosis

In the absence of confirmed fibrosis, the predominant pain mechanism cannot be assumed. This represents a study-level classification rather than a patient-level, fibrosis-unselected phenotype. By including cohorts where fibrosis was unassessed, unconfirmed, or unreported, this category encompasses a broader post-surgical population rather than a homogeneous biologic subgroup. In these populations, persistent pain may reflect a complex interplay of residual structural contributors, neuropathic mechanisms, central sensitization, or pathomechanical dysfunction—factors that were not systematically differentiated at the study level [25,26,27,28,29,30].

Consequently, these findings should not be interpreted as a literal exclusion of clinically relevant epidural fibrosis. Because fibrosis ascertainment is highly method-dependent, this comparator stratum likely reflects differences in diagnostic intensity, study design, or operator expertise rather than a pure biologic absence of the condition.

### 1.4. Epidural Adhesiolysis: A Proposed Multi-Modal Structurally Directed Strategy

The biologically active structural character of epidural fibrosis has direct therapeutic implications, because some interventions attempt to modify the lesion itself rather than merely modulate pain perception downstream. In this framework, EA is aligned with structural pathology, because it is intended to disrupt adhesions, improve target-site access, restore nerve-root mobility, and deliver medication directly to the scar-nerve interface. Specifically, the procedure combines mechanical disruption with the delivery of three pharmacologic agents (hyaluronidase, corticosteroid and hypertonic saline), each contributing a distinct therapeutic mechanism [31,32,33].

**Figure 2:**
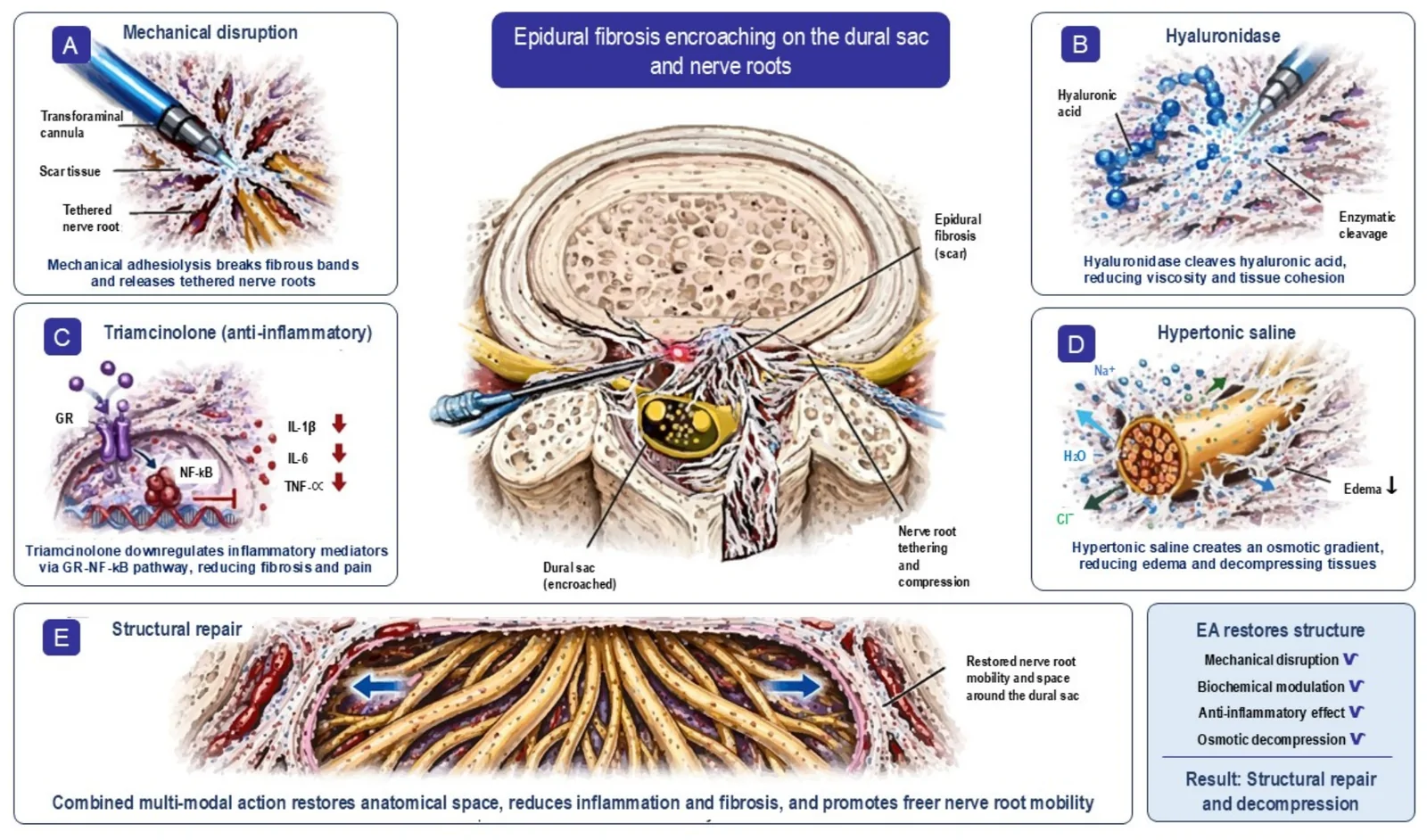
**Epidural adhesiolysis as a proposed multimodal, structure-guided strategy**. A composite schematic illustration of the four complementary mechanisms proposed for epidural adhesiolysis, based on preclinical findings. (**A**) Mechanical disruption of adhesions; (**B**) enzymatic reduction of a hyaluronic acid-rich matrix; (**C**) anti-inflammatory and anti-fibrotic corticosteroid effects; and (**D**) osmotic and neurophysiologic effects of hypertonic saline. (**E**) Structurally directed logic of adhesiolysis [31,32,33]. The schematic depicts a mechanistic rationale and not a pathway demonstrated in the included studies. GR = glucocorticoid; NF-kβ = nuclear factor kappa; IL = interleukin; TNF-∝ = tumor necrosis factor alpha; Na^+^ = sodium; Cl^−^ = chloride; H_2_O = water; ↓ decrease. Figure created by the authors.

These mechanisms are hypothesized to act as a multimodal, structurally directed strategy: mechanical disruption of adhesions, enzymatic degradation of the fibrotic matrix, anti-inflammatory suppression of ongoing fibrosis, and osmotic decompression of neural elements (Figure 2). These combined effects are thought to improve target-site access and nerve-root mobility, although scar reformation and variability in long-term durability remain recognized clinical realities.

### 1.5. Neuromodulation Without Structural Repair: A Hypothesis

**Figure 3:**
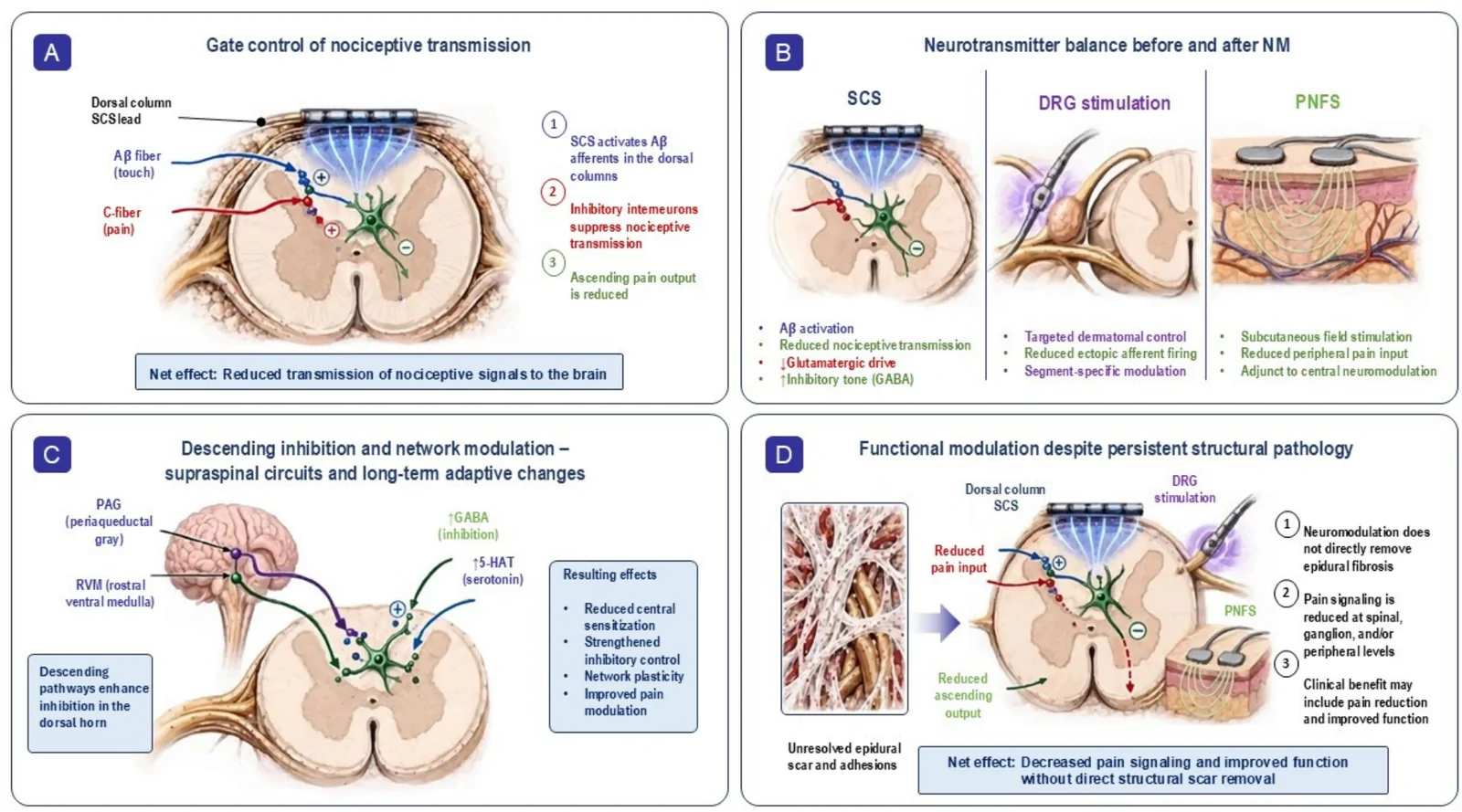
**Pre-clinical neuromodulation mechanisms**. Schematic illustration of neuromodulation mechanisms of pain inhibition: (**A**) Gate control through Aβ-fiber activation and dorsal horn inhibitory interneurons. (**B**) Reduced excitatory transmission before and after stimulation. (**C**) Neuroplasticity and descending inhibitory modulation involving PAG/RVM pathways and inhibitory neurotransmission. (**D**) Persistent structural scar with functional modulation of nociceptive transmission, emphasizing that SCS modulates pain without resolving structural fibrosis [34,35,36,37,38,39,40]. Figure created by the authors. The schematic depicts a mechanistic rationale drawn from preclinical literature and is not a pathway demonstrated by the included studies.

Unlike adhesiolysis, NM is not designed to physically remove or alter epidural scar tissue; its rationale rests instead on altering how pain signals are transmitted and processed at the spinal and brain levels. Since its introduction by Shealy and colleagues [34], spinal cord stimulation (SCS) has worked by delivering electrical current to the dorsal columns, engaging large-diameter Aβ fibers. The gate control framework proposed by Melzack and Wall [35] holds that this Aβ activation dampens incoming nociceptive signals at the dorsal horn through GABA- and glycine-releasing interneurons [36]. Put simply, SCS uses controlled electrical pulses to interrupt or blunt pain signaling before it reaches higher brain centers. This mechanistic basis helps explain why NM can be effective across broad postoperative pain populations even without confirmed fibrosis, since its benefit does not hinge on physically clearing scar tissue or freeing a tethered nerve root. Newer stimulation paradigms—tonic, burst, and 10 kHz high-frequency—appear to add further effects, including long-term depression of excitatory synaptic activity and strengthened descending inhibitory control [37,38], likely via changes in glutamatergic signaling through AMPA and NMDA receptor pathways [39,40]. Ultimately, this functional form of pain modulation leaves structural tethering unresolved, marking a clear mechanistic contrast with the structural-repair approach of adhesiolysis (Figure 3).

### 1.6. Structural Confirmation of Fibrosis: Imaging and Procedural Techniques

Accurate structural confirmation of epidural fibrosis is essential if fibrosis is to be evaluated as a candidate stratifier of treatment response rather than treated as a speculative explanatory construct. A diagnosis depends on multimodal structural confirmatory procedures, including contrast-enhanced MRI, epidurography, epiduroscopy, computed tomography (CT)/myelography and direct intraoperative visualization. Contrast-enhanced MRI remains the most familiar noninvasive postoperative technique, particularly for distinguishing enhancing scar from nonenhancing recurrent disc material [13,41]. A critical interpretive premise is that studies lacking confirmed fibrosis cannot be assumed to represent truly fibrosis-absent populations.

Patient-level fibrosis stratification has not been feasible in previous comparative work. The included studies report fibrosis status at cohort level rather than for individual participants; ascertainment methods differ between studies and are not uniformly reported; individual-participant data were not available; and no comparative trial has prospectively stratified randomization by structurally confirmed fibrosis. Classification at study level is therefore a constraint imposed by the evidence base rather than an analytic preference.

### 1.7. Study Objectives and Hypothesis

These biological and therapeutic distinctions motivate a testable hypothesis: that treatment-response patterns in PSPS-T2 may differ according to whether epidural fibrosis is present, such that structurally directed interventions (EA) align most closely with structurally confirmed fibrosis and functionally directed interventions (NM) align most closely with broader post-surgical populations. This is regarded as a hypothesis to be tested by future prospective work rather than one that can be confirmed by the existing literature. The objectives of the present review were therefore threefold: (1) to synthesize, within fibrosis-defined strata, the comparative effects of EA and NM variants against control; (2) to map the joint distribution of treatment family and fibrosis status across the evidence base, so as to define which comparative questions the literature can and cannot answer; and (3) to examine, as an explicitly exploratory analysis, whether study-level fibrosis status is associated with treatment-effect magnitude, recognizing in advance that fibrosis status and treatment family may be confounded.

## 2. Materials and Methods

### 2.1. Study Design and Registration

The present study constitutes the first part of a multi-stage evaluation stemming from a comprehensive systematic review. This review and network meta-analysis was prospectively registered with PROSPERO (CRD420261291928) and conducted according to PRISMA-NMA guidelines [42]. The original registered review framework was broader and addressed comparative interventional evidence in PSPS-T2/FBSS rather than fibrosis as an a priori stratification factor. The fibrosis-focused framing presented in this manuscript emerged during mature dataset review and manuscript-level specialization and was therefore interpretive and exploratory rather than the sole original registered objective. Protocol-related refinements, terminology changes, comparator clarifications, study-level consolidation rules, and sensitivity logic are documented in Supplementary File S1.

### 2.2. Search Strategy

A systematic search was performed in Medline, PubMed, EMBASE, Cochrane Central Register of Controlled Trials, and Scopus from database inception to 8 February 2026, as well a complementary citation search in Research Gate and Google Scholar (citation and research group tracking). The review included randomized controlled trials (RCTs) (tier 1), prospective cohort studies (tier 2) and retrospective studies (tier 3). No language restrictions were applied. The search strategy (Boolean logic) included various combinations of keywords related to failed back surgery syndrome OR postlaminectomy syndrome OR persistent spinal pain syndrome AND neuromodulation OR spinal cord stimulation OR epidural adhesiolysis OR fibrosis. Full search strings, including the specific Boolean operators, are provided in in Supplementary File S1. The Cochrane Central Register of Controlled Trials indexes records derived from trial registries as well as from bibliographic databases and registry-derived records were therefore screened as part of the primary search. A dedicated systematic search of grey literature—conference abstracts, dissertations, and regulatory agency documents—was not performed, and eligibility was restricted to peer-reviewed publications.

### 2.3. Eligibility Criteria

Studies were included if they: (1) enrolled adults (≥18 years) with PSPS-T2/FBSS and/or epidural fibrosis; (2) evaluated NM or EA; (3) reported pain outcomes (VAS/NRS) or functional outcomes (ODI); and (4) provided data for effect size calculation. Exclusion criteria included: (1) patients with no history of surgery or fibrotic causes of radicular pain; (2) treatments outside the spine; (3) multiple treatments carried out simultaneously or in a crossover design; and (4) single-center retrospective case series (<100 patients), reviews, editorials, case reports and conference contributions.

### 2.4. Study Selection and Data Extraction

Two reviewers independently screened titles, abstracts, and full texts. Disagreements were resolved by consensus or third reviewer. Data were extracted using standardized forms: study characteristics (author, year, country, design), patient demographics, intervention details, fibrosis confirmation method, and outcomes. The EA and NM abbreviations used are listed in Table 1.

### 2.5. The Critical Distinction: EA vs. EA_Std

A methodological nuance critical to this analysis is that many randomized controlled trials of EA include a control arm receiving epidural steroid injection without mechanical adhesiolysis. We designate this as standard EA (EA_Std)—representing the control condition, not true EA (Table 1). In our primary analysis, EA_Std arms are excluded from the EA treatment group. Only studies or study arms that utilize epidural lysis of adhesions are classified as EA.

### 2.6. Classification and Description of Active Treatment Options

Mechanical Adhesiolysis (EA_Mechanical): Under fluoroscopic or endoscopic guidance, a steerable catheter (2.1 mm diameter preferred) is advanced into the epidural space. The catheter is manipulated through fibrotic tissue, physically disrupting adhesions that tether nerve roots [43]. This mechanical lysis restores nerve root mobility—a critical determinant of functional capacity.

Hyaluronidase (EA_Hyal): Hyaluronidase might act as a hydrolytic enzyme that could depolymerize hyaluronic acid, potentially reduce tissue viscosity and hypothetically facilitate steroid penetration [44]. This response assumes hyaluronidase might degrade scars, downregulate TGF-β1, and synergize with mechanical lysis to disrupt adhesion [20].

Corticosteroid (EA_Std): Corticosteroids have proven molecular anti-inflammatory pathways, but their ability to reduce fibrosis is largely hypothetical and clinically unsupported. While cellular pathways like nuclear factor-κB (NF-κB) suppression are well-established [45], clinical evidence shows monotherapy fails to halt progressive fibrotic diseases [15].

Hypertonic Saline (EA_HyperSal): Hypertonic saline likely contributes through osmotic and neurophysiologic mechanisms, specifically by reducing perineural edema and exerting differential nerve conduction blocks. This action preferentially affects small unmyelinated C-fibers over larger myelinated fibers while restoring impaired microcirculation and relieving neural compression fibers [46,47,48].

Conventional low-frequency stimulation (SCS_LF) is categorized as a purely functional modality. Unlike structural repairs, its efficacy relies on the segmental and supraspinal modulation of nociceptive input [34,35]. By leveraging both classical gate-control mechanisms and the recruitment of descending inhibitory pathways, SCS_LF addresses pain at the level of neural processing [36].

Burst stimulation (SCS_Burst) represents a modified neuromodulatory strategy intended to alter pain processing with less reliance on continuous paresthesia. Mechanistically, burst-pattern stimulation is thought to influence both segmental nociceptive gating and broader affective-attentional dimensions of pain processing, thereby extending NM beyond simple tonic dorsal-column activation [36,38].

High-frequency and subperception stimulation (SCS_HF10/SCS_SUBP) are best understood as non-structural pain-modulation strategies. While their effects depend less on classic paresthesia-linked gating and more on the modulation of excitatory–inhibitory balance within spinal circuits, they remain mechanistically aligned with the broader NM family. This alignment holds even if the specific waveform delivery differs significantly from conventional low-frequency or burst protocols [36,37,49].

Dorsal root ganglion stimulation (DRG) represents a more segmentally focused neuromodulatory approach, targeting sensory processing closer to the affected dermatome rather than relying on broader dorsal-column field effects. Conceptually, DRG remains within the same functional-modulation family; its therapeutic logic seeks to alter afferent pain signaling and segmental hypersensitivity [4,50].

Peripheral nerve field stimulation and related subcutaneous approaches (SCS_PNFS) extend NM beyond the dorsal columns by targeting nociceptive input more peripherally within the painful field itself. PNFS remains a functional-modulation strategy, often serving as an adjunct to conventional SCS rather than a structural intervention. Its inclusion in the NM family is justified by its shared therapeutic goal [51,52].

### 2.7. Fibrosis Classification

Studies were classified at the study level as fibrosis-positive when the analyzed cohort or subgroup was explicitly described as having structurally confirmed epidural fibrosis via one or more direct assessment methods reported in the study, including contrast-enhanced MRI [13], epidurography [53,54], epiduroscopy [12], CT/myelography, intraoperative confirmation, or equivalent procedure-relevant structural documentation [55]. A single explicit structural confirmation method was sufficient for fibrosis-positive classification. Studies were classified as studies without confirmed fibrosis (unselected) when fibrosis was not structurally assessed, not explicitly reported, or not required for eligibility. This category was not interpreted as proving absence of fibrosis. Where studies used mixed or partially specified ascertainment methods, classification favored the most explicit structurally documented study-level phenotype available (fibrosis-positive) in the publication set.

Classification was necessarily performed at study level rather than for individual participants: individual-participant fibrosis data were not available from the included reports, and ascertainment methods were not uniformly described across studies. Study-level classification therefore reflects a constraint imposed by the available evidence rather than an analytic preference, and its interpretive consequences are considered in the Limitations.

### 2.8. Quality Assessment

Risk of bias was assessed using the Cochrane Risk of Bias 2.0 tool for RCTs [56] and the ROBINS-I tool for non-randomized and retrospective cohort studies [57]. Studies were categorized as having low, moderate, serious, or critical risk of bias. Potential publication bias and study-level heterogeneity were investigated using contour-enhanced comparison-adjusted funnel plots, and Egger’s test was applied to statistically evaluate asymmetry. To evaluate the stability and robustness of the pooled network meta-analysis estimates, a sensitivity analysis was conducted by restricting the model to include only RCTs (tier 1) and, separately, RCTs + non-RCTs (tier 1 + 2). These results were compared with the primary network analysis (which included all studies) to determine the influence of the retrospective data.

### 2.9. Outcome Measures

This study recorded all defined post-treatment outcomes at 3, 6, 12, and 24 months for RCTs, and at the longest available time point after treatment for non-randomized studies. Primary outcomes were as follows: (1) Pain intensity, measured on a 0–10 cm Visual Analogue Scale (VAS) or 0–10 Numeric Rating Scale (NRS) capturing average pain. If both back and leg pain were recorded, the pain corresponding to the patient’s predominant symptom was selected. (2) Functional disability, measured by the Oswestry Disability Index (ODI, 0–100%) [58]. When nonstandard functional disability measures were reported, harmonized directional transformations were performed where methodologically justifiable to permit inclusion in pooled comparative analyses. Secondary outcome was the responder outcome, defined as the proportion of participants achieving a clinically meaningful reduction of ≥50% in pain intensity from baseline to post-intervention. Where non-standard functional instruments were reported, directional harmonization preserved relative improvement rather than assuming numerical equivalence between scales, and responder analyses combined studies using ≥50% and ≥30% thresholds. These transformations introduce uncertainty in a predictable direction: harmonized disability values should be read as approximate rather than as true ODI measurements, and inclusion of the lower responder threshold may modestly inflate pooled response rates while contributing to heterogeneity. Because only a small number of studies required either transformation, the influence on pooled estimates is expected to be modest; this is nevertheless flagged as a source of uncertainty in the Limitations.

### 2.10. Network Meta-Analysis and Evidence Geometry

Primary network analyses were performed using a frequentist random-effects framework in R using the netmeta package (RSTUDIO, Version: 2026.01.2+418), which synthesizes direct and indirect evidence within a unified graph-theoretical approach [59]. Because the retained subgroup networks differed materially in their available comparator structures, family-level estimates were interpreted within strata rather than as direct cross-stratum comparative-effectiveness estimates. Fibrosis status was assigned at the study level. Consequently, family-level groups were stratified as positive for studies with confirmed fibrosis and unselected for those where fibrosis was not assessed or confirmed. Studies were included in the network meta-analysis only if they documented the comparative efficacy of at least two treatments (including a control group). Different technical approaches to the same treatment were not considered as separate comparison groups in this analysis.

Multi-arm studies were handled within the network framework so that within-study correlations were preserved rather than treated as independent pairwise observations. Where a single trial had generated more than one publication, these were consolidated into one study family so that the cohort entered the analysis once; the selection across follow-up timepoint per study is described in Section 2.11. Treatment ranking was summarized using P-scores, which were interpreted as exploratory ranking summaries rather than as definitive comparative-effectiveness evidence. To improve transparency, Supplementary Materials report network geometry, node-splitting inconsistency outputs, heterogeneity statistics, and additional sensitivity analyses categorized by level of evidence. Where available, the supplement also reports the proportion of direct versus indirect evidence contributing to key subgroup estimates. This review used a frequentist exploratory network meta-analysis framework with substantial clinical and methodological heterogeneity across evidence tiers and fibrosis-stratified subgroup architectures.

### 2.11. Transitivity, Comparator Structure, and Follow-Up

Transitivity was assessed before synthesis. We examined how potential effect modifiers were distributed across comparisons: baseline pain and disability, age, sex, fibrosis ascertainment method, comparator structure, and study design. Baseline pain did not differ significantly between the treatment families and control (*p* > 0.05). Baseline disability did differ (*p* < 0.05), indicating some imbalance at entry, and both age and sex acted as significant moderators in meta-regression (*p* < 0.001). The decisive finding, however, concerned the network itself. Treatment family and fibrosis status are confounded in this dataset, and the two subgroup networks share no active treatment node. Transitivity is therefore not satisfied for any cross-family comparison: an EA-versus-NM contrast would rest on the untestable assumption that the two strata are exchangeable. Quantitative synthesis was accordingly restricted to within-family, within-stratum comparisons against a common control. Where necessary, the cross-family effect was computed.

Each study contributed a single observation to each network. Where a trial reported outcomes at several follow-up intervals, the timepoint closest to the prespecified primary window of six months was selected; where two intervals were equidistant, the earlier was taken. Linked publications reporting different follow-up timepoints from the same underlying trial were consolidated at study-family level, so that no cohort contributed more than once to any analysis. The timepoint retained for each study is recorded in the supplementary duplication audit. Restriction to Tier 1 and to Tiers 1 + 2 were analyzed separately as sensitivity analyses rather than pooled with the primary window.

### 2.12. Interaction Testing

Because study-level fibrosis status was largely determined by which treatment a study evaluated, a meta-regression using fibrosis status as a moderator cannot in principle separate the influence of fibrosis from that of treatment family; the analysis is therefore reported strictly as an exploration of heterogeneity. Exploratory interaction testing was used to examine whether treatment-response patterns differed according to study-level fibrosis-confirmation status. This interaction analysis was exploratory and distinct from the primary within-stratum family-level network summaries. A mixed-effects meta-regression using fibrosis status as a study-level moderator was performed to assess whether fibrosis status explained treatment-effect variability across the broader evidence base. The omnibus test of moderators (QM) was used to assess subgroup differences, and Cochran’s Q-test (QE) was evaluated to measure residual heterogeneity. If the remaining heterogeneity is high and the comparative structures differ across the various subgroup networks, the moderator analysis will be regarded as supporting evidence and not as the sole criterion for the central hypothesis of this study.

## 3. Results

### 3.1. Study Selection and Characteristics

**Figure 4:**
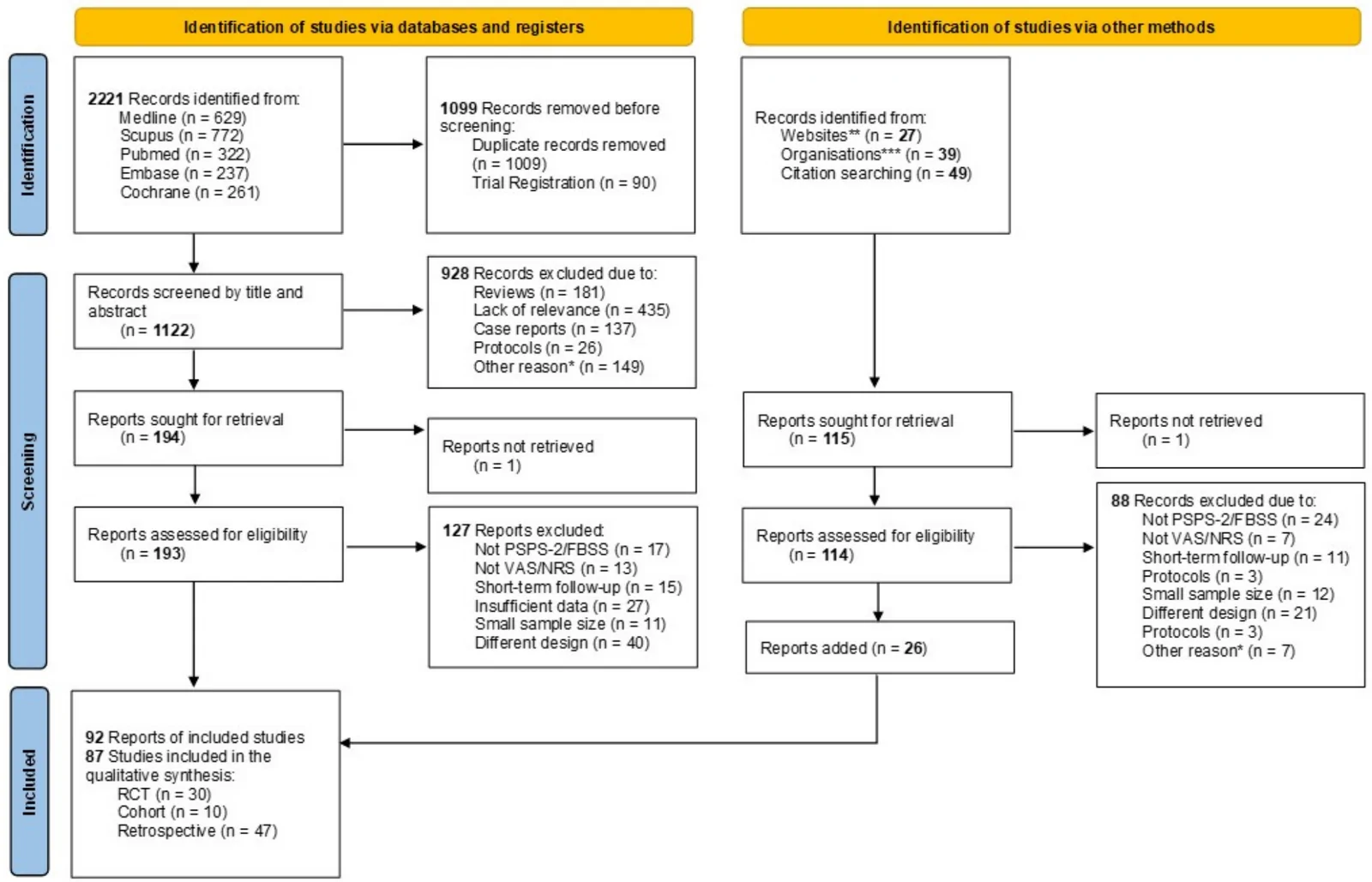
**PRISMA 2020 flow diagram of studies through the review process**. * Conference contributions, letters, editorials; ** Google Scholar, ResearchGate; *** Mohammed Bin Rashed University of Medicine and Health Sciences Library, Dubai and Khalifa University Library, Abu Dhabi, UAE.

From 2221 records identified, 1122 remained after removing duplicates and initial screening. Ultimately, 87 studies (92 publications) involving 33,504 patients met inclusion criteria (Figure 4). This total comprised 30 RCTs (n = 2400), 10 prospective cohorts (n = 901), and 47 retrospective cohorts or registries (n = 30,203). Across the database, 40 studies (n = 4180) were classified as fibrosis-positive and 47 (n = 29,324) as fibrosis-unselected. EA treatments were more prevalent in the fibrosis-positive group, while NM treatments dominated the fibrosis-unselected group. Only one non-randomized study has performed a direct, head-to-head comparison evaluating the treatment effects of Dorsal Root Ganglion (DRG) NM versus EA with hyaluronidase [59]. Only one study reporting the use of the SCS_SUBP treatment could be included in the meta-regression analysis [60].

As shown in Table 2, the composition of study designs was balanced across both groups. Within RCTs, 16 studies (n = 1055) were fibrosis-positive compared to 14 (n = 1345) without confirmed fibrosis. Prospective cohorts were split evenly (5 studies each, n = 311 positive; n= 590 unselected), while retrospective studies included 19 fibrosis-positive (n = 2814) and 28 fibrosis-unselected (n = 27,389) cohorts. This distribution suggests that confounding with treatment family precludes attributing this signal to fibrosis itself (Supplementary File S2). Among all trials, the higher patient count in the fibrosis-unselected subgroup likely stems from differences in study design and ascertainment practices, rather than providing evidence that fibrosis was absent in these larger cohorts.

Methodological inconsistencies across the included literature present a challenge to synthesis. These include diverse justifications for treatment designs, non-standardized outcome criteria, and clinical variations in study populations (Supplementary File S1). Studies containing mixed chronic lumbar pain populations were retained when postoperative PSPS-T2/FBSS patients represented a clinically relevant component of the cohort and the intervention mechanism remained applicable to the postoperative fibrosis-associated setting. The EA_HyperSal node included protocols using hypertonic saline as well as selected high-volume saline adhesiolysis approaches when saline-mediated hydrodissection or osmotic augmentation formed part of the intended treatment mechanism.

### 3.2. NMA Results: Primary Within-Stratum Pain Results Versus Control

**Figure 5:**
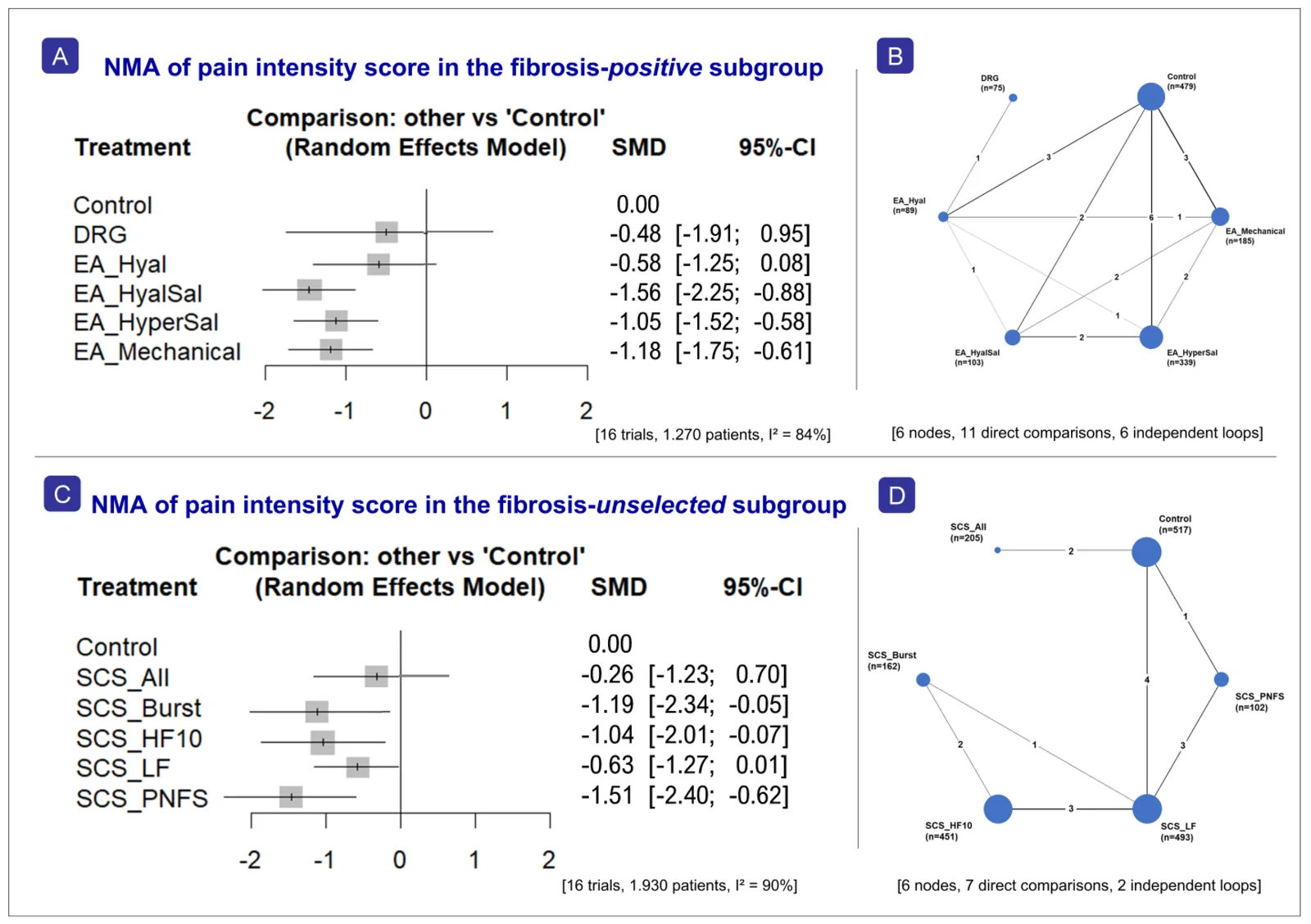
Network meta-analysis of pain intensity score (0–10 cm). (**A**) Forest plot for standardized mean differences of treatment options on pain intensity scores for studies in the fibrosis-positive subgroup. (**B**) Geometry for fibrosis-positive network. (**C**) Forest plot for standardized mean differences of treatment options on pain intensity scores for studies in the fibrosis-unselected subgroup. (**D**) Geometry for fibrosis-unselected network. Each study contributes one observation per network, at the follow-up timepoint nearest six months. DRG and SCS_All appear in the primary networks but exist only in Tiers 2–3. The number displayed on each line indicates the total comparison count between two specific nodes.

This network meta-analysis shows a mixed pattern of effectiveness across treatments when compared with control (EA_Std, conventional medical management-CMM, placebo/sham), as indicated by standardized mean differences (SMD) (Figure 5).

The analysis yields two implications that are crucial for the interpretation of all the results presented below. Firstly, whilst the within-family and within-stratum networks (Section 3.2, Section 3.3 and Section 3.4) are internally valid, they describe different populations and cannot be combined into a single ranking. Secondly, given the high heterogeneity of the networks (Section 3.5), the structural dependence should lead to a downgrading of the certainty of the evidence. The full list of studies included in the NMA is set out in Supplementary File S2.

#### 3.2.1. Fibrosis-Positive Subgroup

This frequentist network meta-analysis (random-effects model; 16 trials, 1270 patients) indicated that every point estimate favored active treatment over control (negative SMDs) (Figure 5A), with EA_HyalSal, EA_HyperSal, and EA_Mechanical yielding the largest effects (all *p* < 0.001) and showing the most consistent and statistically significant effects. EA_Hyal (*p* = 0.085) and DRG (*p* = 0.508) did not differ significantly from control. Among active treatments, EA_HyalSal tends to perform better than EA_HyperSal and EA_Hyal, while differences between EA_HyperSal and EA_Mechanical are small and non-significant.

The network plot in this subgroup contains six treatment nodes—the largest NMA network in this analysis—with 11 direct comparisons forming a partially connected hub-and-spoke structure, as well as additional edges creating 6 closed loops (Figure 5B). Comparisons involving DRG rest on a single direct edge and are imprecise, with wide confidence intervals crossing zero, indicating no clear differences. Substantial heterogeneity (I^2^ = 83.7%, 95% CI 74.5–89.6; τ^2^ = 0.36) and significant between-design inconsistency (Q = 42.83, 9 df, *p* < 0.001) indicate that study-design variation influences the outcomes. Node-splitting nevertheless identified no individual contrast at which direct and indirect estimates disagreed significantly, so the incoherence is distributed across designs rather than localized to one comparison (Supplementary File S2).

In the fibrosis-positive supportive network, the probability score reveals EA_HyalSal as the most effective treatment (Pscore = 0.986), while control group ranks last (Pscore = 0.033) (Supplementary File S2) These are summaries of probability rankings and do not constitute evidence of any comparative superiority. The comparison-adjusted funnel plot for this network is visually asymmetric, but the asymmetry is not statistically supported by Egger’s test (*p* = 0.45). While larger, more precise studies cluster closer to the center, smaller studies tend to report more extreme effects, suggesting that the overall treatment effect may be somewhat overestimated.

#### 3.2.2. Fibrosis-Unselected Subgroup

For the fibrosis-unselected subgroup (16 trials, 1930 patients), the network meta-analysis indicates that all NM modalities reduce the outcome compared to control (negative SMDs) (Figure 5C). Among the interventions, SCS_PNFS appears the most effective (largest negative SMD), followed by SCS_Burst and SCS_HF10. SCS_Burst, SCS_HF10, SCS_LF, and particularly SCS_PNFS demonstrate statistically significant benefits (*p* < 0.05), while SCS_All shows a non-significant effect, with its confidence interval crossing zero (*p* = 0.092). The network plot reveals a connected evidence structure of six nodes joined by 7 distinct direct comparisons, with SCS_LF acting as the principal connector (Figure 5D); two independent closed loops are present. Control versus SCS_HF10 and control versus SCS_Burst carry no direct evidence and are estimated indirectly, with correspondingly wide intervals.

Heterogeneity in this network is substantial (I^2^ = 89.6%, 95% CI 83.8–93.4; τ^2^ = 0.45) (Supplementary File S2). Both within-design heterogeneity (Q = 96.37, 9 df, *p* < 0.001) and between-design inconsistency (Q = 9.78, 2 df, *p* = 0.008) are statistically significant, suggesting that differences in study design and disagreement between direct and indirect evidence affect the robustness of the estimates. The netsplit analysis confirms significant inconsistencies, most notably in the SCS_HF10:SCS_LF and SCS_Burst:SCS_HF10 comparisons, where direct and indirect estimates disagree significantly. Additionally, several direct comparisons show very high heterogeneity (I^2^ 78.8–97.5%).

The funnel plot shows pronounced asymmetry, suggesting significant heterogeneity or publication bias (Supplementary File S2). Egger’s test on the comparison-adjusted estimates is not significant (*p* = 0.085). The probability score test places SCS_PNFS first (Pscore =0.823) and control last (Pscore =0.009). These are exploratory summaries, and not evidence of comparative superiority (Supplementary File S2).

Taken together, these results indicate a differential pattern of within-stratum treatment-response behavior across fibrosis-defined study strata, but they do not constitute definitive evidence of EA-versus-NM comparative superiority within either stratum. Because cross-family EA-versus-NM comparisons were predominantly indirect, node-level rankings and representative time-window signals were treated as supportive rather than sovereignty-bearing analyses.

### 3.3. NMA Results: Primary Within-Stratum ODI Results Versus Control

This network meta-analysis (12 trials) shows a mixed pattern of effectiveness across treatments compared with control (EA_Std, CMM, placebo/sham)—specifically, although results varied, most evaluated treatments produced a significant improvement in ODI.

#### 3.3.1. Fibrosis-Positive Subgroup

**Figure 6:**
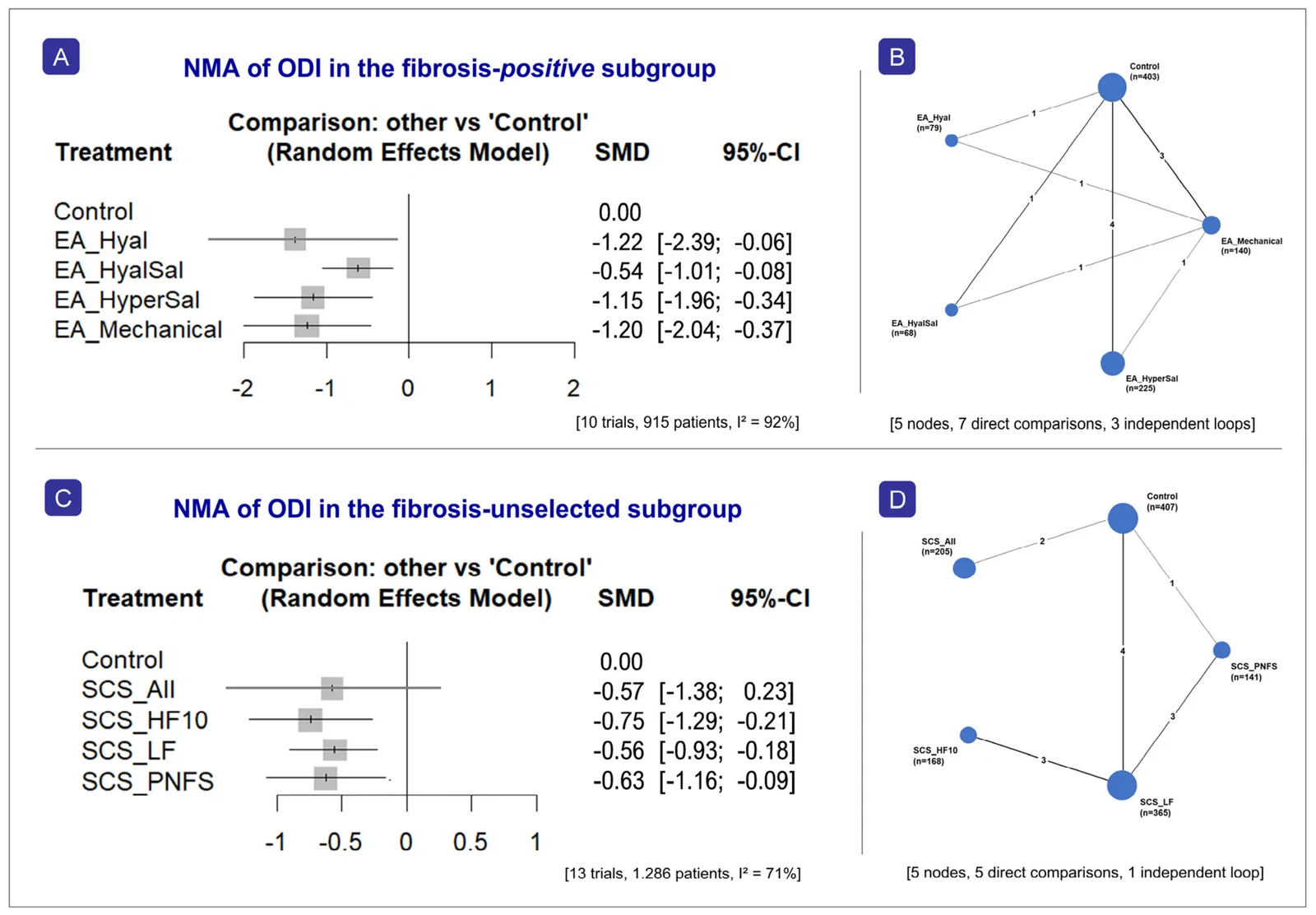
Network meta-analysis of ODI. (**A**) Forest plot for standardized mean differences of treatment options on ODI for studies with confirmed fibrosis. (**B**) Geometry for fibrosis-positive network. (**C**) Forest plot for standardized mean differences of treatment options on ODI for studies in the fibrosis-unselected subgroup. (**D**) Geometry for fibrosis-unselected network. Each study contributes one observation per network, at the follow-up timepoint nearest six months. SCS_All appears in the primary networks but exists only in Tiers 2–3. The number displayed on each line indicates the total comparison count between two specific nodes.

The analysis (10 trials, 915 patients) shows that all evaluated treatments (EA_Hyal, EA_HyalSal, EA_HyperSal, and EA_Mechanical) significantly improve ODI compared with the control group, as indicated by negative SMD values (Figure 6A). Among them, EA_Hyal demonstrates the largest effect (*p* = 0.05), followed closely by EA_Mechanical (*p* = 0.005) and EA_HyperSal (*p* = 0.005), while EA_HyalSal shows a comparatively smaller effect (*p* = 0.05). The network plot for the fibrosis-positive subgroup comprises five nodes joined by 7 distinct direct comparisons (Figure 6B). Control is the busiest node, but direct EA-versus-EA evidence is also present, linking EA_Mechanical to each of EA_Hyal, EA_HyalSal and EA_HyperSal. The network contains 3 independent closed loops.

Heterogeneity across this network is the highest of the six primary networks (I^2^ = 91.9%, 95% CI 86.5–95.2; τ^2^ = 0.67). Both the within-design heterogeneity test (Q = 19.98, 3 df, *p* < 0.001) and the between-design inconsistency test (Q = 66.74, 4 df, *p* < 0.001) are statistically significant, indicating disagreement between direct and indirect evidence that materially limits the robustness of the comparisons (Supplementary File S2).

The netsplit analysis also show that all treatments outperform the control in the network estimates, with EA_Hyal, EA_HyperSal, and EA_Mechanical demonstrating consistent and statistically significant effects, while EA_HyalSal also shows a benefit, but with some inconsistency between direct and indirect evidence. Notably, for EA_Hyal and EA_HyalSal versus control, the direct and indirect estimates differ in magnitude—and even direction in the case of EA_HyalSal—suggesting potential inconsistency in the network. In contrast, EA_HyperSal and EA_Mechanical versus control display close agreement between direct and network estimates, indicating more reliable evidence (Supplementary File S2).

The funnel plot shows asymmetry, and Egger’s test on the comparison-adjusted estimates is statistically significant (*p* = 0.018), indicating small-study effects or publication bias in this network. The probability score test reveals EA_Hyal as the most effective treatment (Pscore = 0.785), while the control group ranks last (Pscore = 0.000) (Supplementary File S2). The three leading P-scores are best described as indistinguishable rather than ordered (Supplementary File S2).

#### 3.3.2. Fibrosis-Unselected Subgroup

In the fibrosis-unselected subgroup (13 trials, 1.286 patients), the network plot consisted of five treatment nodes: control, SCS_PNFS, SCS_LF, SCS_HF10, and SCS_All (Figure 6C). All active nodes improve ODI relative to control, as reflected by negative SMD values. However, SCS_All does not reach statistical significance. SCS_HF10 shows the strongest and statistically significant effect, followed by SCS_PNFS, SCS_All, and SCS_LF, all not separable from one another. Notably, there was no direct edge connecting control and SCS_HF10, but the network is connected and that contrast is estimable through indirect evidence (Figure 6D)—joined by 5 distinct direct comparisons forming one independent closed loop.

Heterogeneity here is the lowest of the six primary networks (I^2^ = 71.1%, 95% CI 44.9–84.8; τ^2^ = 0.12), indicating moderate-to-high variability in effect sizes (Supplementary File S2). Inconsistency between direct and indirect evidence is minimal: the between-designs test is not statistically significant (Q = 0.13, 1 df, *p* = 0.722), indicating that the network estimates are coherent despite the observed heterogeneity.

The netsplit plot suggests that, for most comparisons, there is no strong evidence of inconsistency between direct and indirect estimates, as their confidence intervals largely overlap, indicating good agreement. Likewise, SCS_PNFS vs. control shows a stronger effect in the indirect estimate, but the network estimate remains consistent and significant. For contrasts such as SCS_HF10 vs. SCS_LF and SCS_LF vs. SCS_PNFS, the confidence intervals cross zero and overlap considerably, suggesting no meaningful differences and acceptable consistency (Supplementary File S2).

The funnel plot shows only slight asymmetry, and Egger’s test computed on the 13 comparison-adjusted estimates is not significant (*p* = 0.715), so there is no statistical evidence of small-study effects in this network (Supplementary File S2). The probability score test places SCS_HF10 first (P-score 0.839), while the control group ranks last (Pscore = 0.022) (Supplementary File S2).

### 3.4. Mixed-Effects Meta-Regression

**Figure 7:**
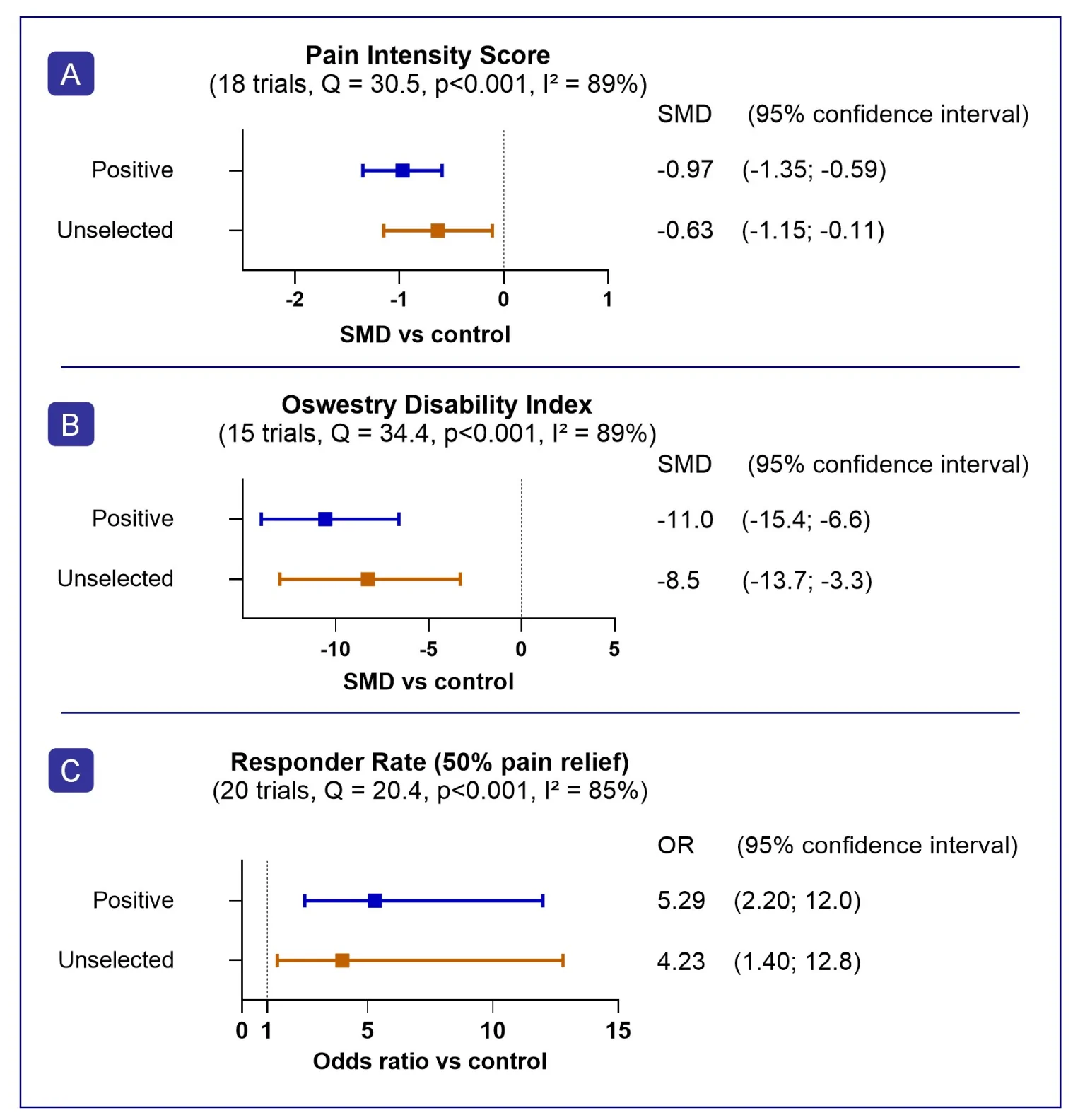
Mixed-effects meta-regression stratified by fibrosis status. Meta-regression results showing: (**A**) significant pain reduction in both the fibrosis-positive and fibrosis-unselected strata (18 trials); (**B**) significant disability (ODI) improvement in both strata, with substantially overlapping intervals (15 trials); (**C**) significantly increased odds of response in both strata (20 trials). Fibrosis status and treatment family are confounded: the moderator indexes which treatment family dominates the evidence in each stratum and does not isolate an effect of fibrosis.

To explore sources of statistical heterogeneity, a mixed-effects meta-regression (categorical subgroup analysis) was performed. Fibrosis status was entered into the model as a moderator. As established in Section 3.2, fibrosis status is confounded with treatment family in this dataset; the moderator terms below therefore index a difference between EA-dominated and NM-dominated evidence and must not be read as isolating an effect of fibrosis. The therapeutic interventions yielded significant improvements across both symptomatic and functional metrics (Figure 7).

Regarding pain outcomes (18 studies, 21 contrasts), results demonstrated substantial residual heterogeneity across the included studies (I^2^ = 89.49%; QE = 204.45, *p* < 0.001), which persisted even after accounting for subgroup classifications; the moderator analysis was significant (QM = 63.74, *p* < 0.0001). Subgroup evaluation confirmed significant pain reductions in both the fibrosis-positive cohorts (pooled effect estimate = −0.9716; 95% CI −1.3538 to −0.5894, *p* < 0.001) and the unselected subgroup (pooled estimate = −0.6322; 95% CI −1.1522 to −0.1121, *p* = 0.017) (Figure 7A). The point estimate is larger in the fibrosis-positive stratum, but the two intervals overlap and, as set out in Section 3.3, the moderator indexes which treatment family dominates each stratum rather than an effect of fibrosis.

The analysis of functional recovery via the ODI (15 studies, 16 contrasts) followed a broadly similar pattern. Marked residual variance was again present (I^2^ = 89.40%; QE = 101.10, *p* < 0.001), indicating substantial between-study differences beyond random chance, and the moderator test was significant (QM = 34.38, *p* < 0.001). In fibrosis-positive studies the pooled effect estimate was −11.01 ODI points (95% CI −15.39 to −6.62, *p* < 0.001); in the unselected it was −8.46 (95% CI −13.65 to −3.28, *p* = 0.001) (Figure 7B). The two strata are separated by 2.5 ODI points with substantially overlapping intervals.

Furthermore, the mixed-effects meta-regression regarding responder rates (50% pain relief) (20 studies, 23 contrasts) demonstrated substantial residual heterogeneity (I^2^ = 85.02%; τ^2^ = 2.121), indicating that a considerable proportion of variability across studies remained unexplained after accounting for the subgroup moderator. This residual heterogeneity test was statistically significant (QE = 156.50 on 21 df, *p* < 0.001), confirming persistent between-study differences, and the moderator test was significant (QM = 20.43 on 2 df, *p* < 0.001). Both strata showed significantly higher odds of response than control—fibrosis-positive OR 5.29 (95% CI 2.20 to 12.69; log-OR 1.6653, *p* < 0.001) and fibrosis-unselected OR 4.23 (95% CI 1.40 to 12.79; log-OR 1.4422, *p* = 0.011)—but the two intervals overlap almost entirely and the strata are not statistically distinguishable from one another (Figure 7C).

### 3.5. Inconsistency and Sensitivity Analyses

Sensitivity analyses (Tier 1 and Tiers 1 + 2) closely matched the all-tier network meta-analysis, showing that including retrospective studies (tier 3) did not alter the main benefit trends for pain and disability. Thirty sensitivity refits were performed: each of the six primary networks was re-estimated at 3-, 12- and 24-month follow-up windows and under restriction to Tier 1 and to Tiers 1 + 2. All 30 converged and no treatment node was dropped by the estimator; the tier-restricted networks contain fewer nodes only because fewer studies contribute to them. The EA_HyalSal node was stable for pain (in all five fibrosis-positive scenarios; SCS_PNFS in all five fibrosis-unselected scenarios) and for responder rates (SCS_PNFS in all five fibrosis-unselected scenarios; EA_HyperSal or EA_Mechanical in the fibrosis-positive scenarios). For ODI, the performing node was scenario-dependent, alternating between EA_Mechanical and EA_HyperSal in the fibrosis-positive stratum and between SCS_HF10 and SCS_PNFS in the fibrosis-unselected stratum; these intervention placements should therefore not be read as a rigid hierarchy.

Heterogeneity varied widely across the refits (I^2^ 22.5% to 92.4%; lowest in the Tier-1-only fibrosis-unselected responder network, highest in the 3-month fibrosis-unselected pain network). A detailed description can be found in the attached file (Supplementary File S2).

### 3.6. Risk of Bias and Publication-Bias Summary

**Figure 8:**
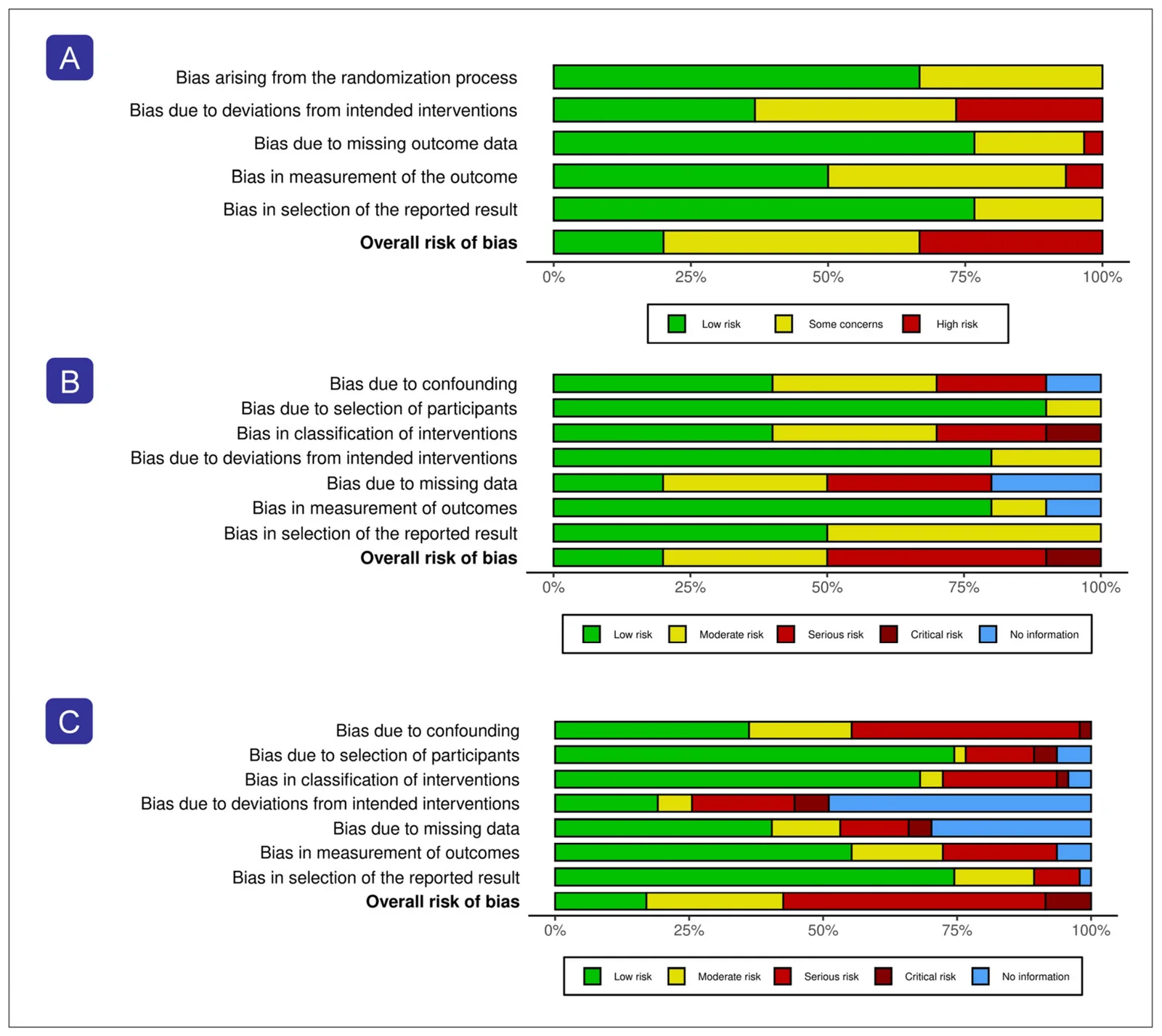
**Study quality assessment (RoB 2 and ROBINS-I).** Risk of bias assessed using: (**A**) the Revised Cochrane Risk-of-Bias Tool for Randomized Trials and (**B**,**C**) the ROBINS-I tool for non-randomized studies (tier 2 + 3). Judgments for RoB 2 domains represent: low risk, some concerns, or high risk. Judgments for ROBINS-I domains represent: low, moderate, serious, or critical risk of bias.

The overall strength of evidence is constrained by methodological limitations—specifically study design, potential selection bias, and inconsistent reporting—which may attenuate the validity of the findings (Figure 8). Therefore, results should be interpreted within these limitations, as they introduce a notable risk of bias and reduce overall certainty. Detailed risk-of-bias assessments (tiers 1–3) and supporting funnel plots are provided in the Supplementary File S2. Table 3 summarizes the key findings by fibrosis stratum of the primary network.

### 3.7. Evidence Limitations

Across comparisons, certainty was rated low to very low. Several domains contributed. Within-study risk of bias was frequently moderate or high, and heterogeneity was substantial to extreme (I^2^ 71–92% across the primary networks and 85–89% across the three meta-regression models). Several within-family contrasts rested on indirect evidence with wide confidence intervals—most notably control versus SCS_HF10 in the fibrosis-unselected ODI network, which carries no direct edge, although it remains estimable—and localized incoherence was present in the fibrosis-positive ODI network, where the EA_HyalSal contrasts against control and against EA_Mechanical yield direct and indirect estimates of opposite sign (side-split *p* = 0.002 for both). No comparison in any of the primary networks was disconnected. Small-study effects were detected in the fibrosis-positive ODI network (Egger *p* = 0.018), but in neither pain network (*p* = 0.449 and *p* = 0.085). Beyond these familiar domains lies a more fundamental constraint. Treatment family and fibrosis status are collinear within the randomized evidence, so even a precise within-stratum estimate cannot support an inference about fibrosis acting independently of treatment (Table 4). The body of evidence is therefore best regarded as hypothesis-generating rather than confirmatory, and the treatment’s probability score reported above as exploratory summaries rather than evidence of comparative superiority.

## 4. Discussion

### 4.1. Principal Findings

The principal finding of this review is structural rather than comparative: in the existing PSPS-T2 literature, treatment family and fibrosis status are almost entirely confounded, and among randomized trials they are perfectly collinear. EA has been studied almost exclusively in structurally confirmed, fibrosis-positive populations, and NM almost exclusively in unselected post-surgical populations. As a result, the two treatment literatures describe different patients, and the field does not yet contain the evidence needed to compare them within a common fibrosis stratum. Within that constraint, the within-family analyses are informative on their own terms: among fibrosis-positive studies the EA variants incorporating hyaluronidase and hypertonic saline achieved the highest probability score for both pain and disability, and among fibrosis-unselected studies peripheral-nerve-field and high-frequency SCS achieved the highest probability scores. What cannot be inferred from these data is that fibrosis itself determines which treatment works better, because in every randomized comparison the two are indistinguishable.

The contribution of this study is therefore to make that confound explicit and to reframe epidural fibrosis, for now, as a biologically grounded candidate stratifier. Positioning fibrosis in this way is not a retreat from the hypothesis but a precondition for testing it: naming the structure of the evidence identifies exactly the trial that would be needed to move fibrosis from plausible to proven.

### 4.2. What the Present Analysis Does and Does Not Show

The present analysis characterizes the architecture of the evidence rather than a treatment hierarchy. Direct EA-versus-NM comparisons within fibrosis-defined strata were, as anticipated, entirely absent. The observed association between fibrosis-positive status and larger effect size is real but confounded: because fibrosis-positive studies are EA studies and fibrosis-unselected studies are NM studies, the same data are equally well described as ‘EA trials showed larger effects than NM trials.’ No analysis of this literature can separate the two, and readers should treat any apparent fibrosis effect as inseparable from a treatment effect. However, this distinction matters. The absence of head-to-head stratified trials limits direct claims, making fibrosis a candidate variable worthy of investigation in future PSPS-T2 trial design.

A central limitation of the present analysis is that the comparator stratum should not be interpreted as a true fibrosis-unselected population. Studies without confirmed fibrosis were defined by lack of structural confirmation, not by demonstrated absence of fibrosis. Given the known discrepancy between epiduroscopic and MRI-based fibrosis detection, it is likely that at least some of these cohorts included patients with clinically relevant but unrecognized fibrosis [12]. This misclassification may dilute a true effect-modification signal, but it may also reflect broader study-level differences in diagnostic intensity, case selection, operator expertise, geography, era, and publication patterns. Accordingly, the studies-without-confirmed-fibrosis stratum is best understood as a heterogeneous non-confirmation category rather than as a biologically pure comparator phenotype.

Interpretation of the fibrosis-positive effect sizes also depends on control composition. Injection-only comparators classified as EA_Std were analytically separated from true adhesiolysis because they do not include mechanical adhesiolysis. However, EA_Std may still be more active than inert sham alone. The magnitude of benefit observed for EA-family interventions in fibrosis-positive studies therefore reflects both treatment activity and the retained comparator structure of that subgroup network, rather than a simple placebo-only contrast.

### 4.3. Biological Rationale for Fibrosis as a Candidate Stratifier

The biological plausibility of the observed pattern rests on the divergent mechanisms of EA and NM, grounded in the molecular pathogenesis of epidural fibrosis and the differing therapeutic targets of structural versus functional treatment. Epidural fibrosis is a biologically active structural lesion. The combination nerve-root tethering, restricted mobility, and persistent profibrotic signaling provides a mechanistic rational for why structurally targeted interventions may perform differently from purely neuromodulatory approaches [22,23,24]. The plausibility of this structural pathway is strengthened by the fact that profibrotic signaling is not static; reactive oxygen species may further amplify TGF-β1-mediated fibroblast activation and matrix deposition. The mechanistic framework remains biologically plausible rather than finally proven.

EA is the interventional strategy most directly aligned with that need. Mechanical adhesiolysis may restore nerve root mobility [33,43], hyaluronidase may degrade hyaluronic acid within the fibrotic matrix and facilitate tissue penetration [20,44], triamcinolone may suppress NF-κB signaling and dampen cytokine-driven fibrosis [15,45], and hypertonic saline is thought to contribute through osmotic and neurophysiologic effects, including reduction in perineural edema and differential susceptibility of unmyelinated fibers in foundational dorsal-root preparations [46,47,48]. Together, these mechanisms make it biologically plausible that structurally dominant fibrosis-positive disease would show a stronger response to structurally directed intervention (Figure 2). Procedural outcome data further suggest that successful postadhesiolysis contrast passage beyond the foramen may act as a correlate of effective structural release and target-site access [142].

By contrast, studies without confirmed fibrosis represent a broader and less structurally resolved population. In these patients, pain is more likely to include altered dorsal horn processing, central sensitization, glial activation, maladaptive neuroplasticity, and impaired descending inhibition [25,26,27,28,29,30]. NM is aligned with this biology because it modulates nociceptive transmission through gate control and network-level functional modulation rather than structural release [35,36,37,38]. In this sense, the observed treatment-response pattern is not only statistically interesting but mechanistically coherent (Figure 3). The statistical supplement does not convert this mechanistic plausibility into definitive universal moderator proof, but it does support the continued testing of a biologically stratified framework.

### 4.4. Functional Recovery and Durability: A Hypothesized Signature

The functional disability findings sharpen the structural interpretation of the manuscript. If adhesiolysis were acting only as a short-lived analgesic adjunct, one would expect weaker or less differentiated downstream disability effects. Instead, the fibrosis-positive signal identified in the pain analysis extended into ODI, and meta-regression showed that the functional treatment effect was greater in fibrosis-positive studies than in fibrosis-unselected studies. This pattern is biologically plausible: if adhesiolysis reduces scar-related tethering, improves nerve-root mobility, and restores access to the structurally relevant epidural target, downstream gains should also be visible in disability domains such as walking, standing, sitting, and self-care. This interpretation is consistent with prior adhesiolysis literature reporting sustained clinical benefit, including later functional improvement, in selected postoperative radicular pain populations [31,33,82]. It is also supported by procedural studies linking favorable postadhesiolysis contrast distribution with better recovery, suggesting that meaningful benefit depends partly on successful structural access rather than drug delivery alone [142]. In the unselected subgroup, several NM modalities were also favorable for ODI versus control, consistent with the broader SCS literature [37,98,126], but the functional signal was smaller than the fibrosis-positive EA-side pattern.

Taken together, these findings align with the structural-versus-functional framework, indicating that structural release could promote functional persistence if fibrosis drives the pain.

### 4.5. Structural Confirmation of Fibrosis and Procedure-Relevant Assessment

Structural confirmation of epidural fibrosis is strongly method-dependent. In a prospective epiduroscopic FBSS cohort, clinically significant fibrosis was far more frequently detected by epiduroscopy than by MRI, suggesting that fibrosis may be substantially underrecognized when conventional imaging alone is used [12]. Procedural techniques such as epidurography and epiduroscopy are especially relevant because they can provide direct or procedure-linked evidence of adhesions and target-site access [143]. They can also demonstrate whether adhesions limit target access and whether meaningful structural release has actually been achieved. In this context, fibrosis confirmation should be understood as structurally grounded and multimodal.

Beyond baseline structural confirmation, epidurography may also provide procedure-relevant information during adhesiolysis itself; in observational lumbar neuroplasty data, postadhesiolysis extraforaminal contrast distribution was associated with better functional outcome, suggesting that final contrast spread may serve as a procedural correlate of successful structural release [142]. Advanced diffusion-based techniques such as diffusion tensor imaging remain of research interest as future biomarker approaches, but they were not the dominant basis for fibrosis classification in the present dataset and should therefore be regarded here as future-oriented rather than clinically established tools [144,145].

### 4.6. Comparison with Previous Literature

The present findings help explain why earlier EA and NM literatures can appear discordant. Major NM trials reported meaningful benefit in broad post-surgical pain populations [49,98,126], whereas many EA studies reported benefit in cohorts enriched for epidural fibrosis or related structural pathology [31,33,81,105]. These findings align with prior postoperative pain literature showing that structurally targeted interventions perform best when scar-related pathology is demonstrable and that NM performs well in broader neuropathic and refractory pain populations [37,82,98]. The present review extends that literature by showing that this apparent discordance is largely an artefact of population selection: the EA and NM evidence bases were built on structurally different patients, so their divergent results reflect who was studied at least as much as what was done. Documenting that separation—rather than resolving it—is what the current evidence permits.

### 4.7. Implications for Current Clinical Practice

A practical question follows from these findings: should structural confirmation of epidural fibrosis influence treatment selection today? On the present evidence, it should not do so by itself. The association between fibrosis-positive status and larger treatment effects cannot be separated from the treatments delivered in those cohorts, and no randomized comparison has tested both families within a single fibrosis stratum. Where structural assessment is already part of routine postoperative evaluation, documenting the presence, extent and location of fibrosis is clinically reasonable and may inform procedural planning—for example, whether adhesiolysis is technically feasible or whether scarring is likely to obstruct lead placement. It is quite another matter to use fibrosis status as a criterion for choosing between adhesiolysis and neuromodulation, and that step is not supported by the current literature. We regard fibrosis-guided treatment selection as a hypothesis for prospective validation rather than as a present standard of care.

### 4.8. Implications for Future Prospective Trial Design

The implications of these findings are substantial. Postoperative pain in PSPS-T2 should not be treated as a single undifferentiated syndrome when structurally relevant fibrosis may be dominant in one subgroup and absent, unassessed, or non-dominant in another.

The clinical relevance of this stratified framework is reinforced by the historically poor and declining success of repeated lumbar surgery in PSPS-T2, with commonly cited estimates of approximately 30% after a second operation, 15% after a third, and 5% after a fourth, and by 5-year cohort data showing only 34% successful outcome after repeated lumbosacral operation [119,146].

A similar caution emerges from fusion-specific data in established PSPS-T2, where only 35% of patients reported good outcome after instrumented fusion, further underscoring the need for better pathology-aligned non-fusion treatment strategies [147]. The need for such trials is strengthened not by a single statistically universal moderator result, but by the convergence of biologic plausibility, structurally grounded ascertainment logic, and directionally coherent stratified treatment-response patterns across the current evidence base.

A rigorous next-step design would incorporate standardized fibrosis assessment using multimodal structural criteria, followed by stratified randomization to EA versus NM with prespecified pain and functional outcomes. Such a design would directly test whether the differential pattern observed here reflects true treatment-effect modification rather than the combined influence of heterogeneous populations, variable fibrosis ascertainment, and indirect evidence structure. If confirmed prospectively, that would move PSPS-T2 management from empiric sequencing toward genuinely mechanism-informed treatment selection.

The present study therefore strengthens the rationale for future fibrosis-stratified prospective trials in which fibrosis assessment is standardized and treatment effects are tested explicitly within structurally defined populations. Such studies are justified not only by mechanistic plausibility but by convergent evidence across pain, ODI, responder, family-level, and moderator analyses. Whether EA produces true structural resolution of epidural fibrosis will require confirmation in those future prospective clinical studies. Future trials should prospectively prespecify fibrosis ascertainment, comparator structure, and follow-up windows in a way that allows direct EA-versus-NM testing within fibrosis-defined strata. Such trials should distinguish confirmed fibrosis, confirmed absence of fibrosis where feasible, and unassessed populations, rather than collapsing all non-positive cases into a single comparator category. Only this design will allow the present framework to be tested directly and determine whether structurally targeted benefit also reflects true structural resolution of fibrosis.

### 4.9. Limitations

Several factors could undermine the validity of these results. First, fibrosis was classified at study level rather than for individual participants, so inferring a patient-level treatment interaction from these data constitutes an ecological inference; aggregated patterns cannot be assumed to hold within studies, and participant-level fibrosis grades were not available from the included reports. Second, network heterogeneity was substantial across the primary and moderator models, so that point estimates should be read as approximate directional summaries rather than as precise comparative quantities. Direct randomized comparisons of EA versus NM within fibrosis-defined strata are unavailable. The stratum of studies without confirmed fibrosis is heterogeneous and likely includes unrecognized epidural fibrosis, as ascertainment was not uniform. Residual heterogeneity persisted in both pain and ODI moderator models. Study design, sampling methods, and variable presentation carry risk of bias. Fibrosis confirmation may also proxy for diagnostic intensity, operator expertise, case selection, and era. Pooling of ODI subgroups was not feasible for all treatment nodes due to sparse data. Additionally, waveform evolution within the NM family and inadequately specified timepoint aggregation contribute heterogeneity that cannot be fully normalized. The dominant limitation, from which most others follow, is the confounding of fibrosis status with treatment family: because only one study provided both treatments within a single fibrosis stratum, this review can map the comparative evidence and quantify within-family effects, but cannot establish fibrosis as an independent treatment-effect modifier. That question is deferred, by necessity, to prospective stratified trials.

## 5. Conclusions

This systematic review and network meta-analysis shows that, in PSPS-T2, the comparative literature on EA and NM is built on structurally different populations: adhesiolysis has been evaluated almost exclusively where epidural fibrosis is confirmed, and NM almost exclusively where it is not, with the two perfectly confounded among randomized trials. Within fibrosis-defined strata, hyaluronidase- and hypertonic-saline-augmented adhesiolysis showed the highest probability of pain relief in the fibrosis-positive studies, whilst peripheral nerve field stimulation and high-frequency stimulation achieved a high probability in the studies without fibrosis selection, but these strata cannot be joined into a valid head-to-head ranking, and the larger effects seen in fibrosis-positive studies cannot be separated from the treatments delivered there. Epidural fibrosis therefore remains a biologically compelling but still unproven candidate for stratifying interventional treatment. Establishing it as a genuine biomarker for treatment stratification will require prospective studies that hold treatment fixed while varying fibrosis status—optimally, stratified randomization to EA versus NM following standardized multimodal fibrosis assessment. Defining that trial is the most useful contribution the present evidence base can make.

## Figures and Tables

**Table 1:** Description of the abbreviations used in the NMA for neuromodulation, epidural adhesiolysis, and control treatment.

Treatment Node	Category	Definition
EA_Hyal	Epidural Adhesiolysis	EA with steroid with hyaluronidase 1500 IU with/0.9% saline
EA_HyperSal	Epidural Adhesiolysis	EA with steroid with hypertonic saline 10%
EA_HyalSal	Epidural Adhesiolysis	EA with steroid with hyaluronidase with hypertonic saline 10%
EA_Mechanical	Epidural Adhesiolysis	Mechanical Adhesionlysis with epiduroscopy/Catheter
SCS_LF	Neuromodulation	Traditional Low-Frequency SCS (30 < 60 Hz)
SCS_SUBP	Neuromodulation	SCS Subperception (500 < 1200 Hz)
SCS_HF10	Neuromodulation	10 kHz High-Frequency
SCS_Burst	Neuromodulation	BurstDR™
SCS_PNFS	Neuromodulation	SCS and peripheral nerve field stimulation/subcutaneous
DRG	Neuromodulation	Dorsal Root Ganglion
SCS_All	Neuromodulation	All SCS * Category
EA_Std	Control	ESI with dexamethasone-hydrophil/with triamcinolone-particulate/steroid with 0.9% saline
CMM	Control	Conventional medical management
Placebo	Control	Sham procedure/placebo

* SCS: Spinal Cord Stimulation.

**Table 2:** **Selected articles and characteristics (Tier 1 + 2 + 3).** Epidural adhesiolysis (EA) and neuromodulation (NM).

First Author	Year	Design	N	Treatment	Fibrosis Status	Confirmation	Months
Akbas M [61]	2018	RCT	60	EA	Positive	MRI/Epidurography	6
Akbas M [62]	2019	Retrospective	120	NM	Unselected		3
Akbas M [63]	2020	Retrospective	148	EA	Positive	Epiduroscopy	12
Bayerl S [64]	2024	Retrospective	154	NM	Unselected		19
Bernaerts L [65]	2024	Retrospective	6170	NM	Unselected		6
Bolash R [66]	2019	RCT	99	NM	Unselected		6
Bretherton B [50]	2022	Retrospective	374	NM	Unselected		24
Burton CV [67]	1977	Retrospective	198	NM	Unselected		24
Campwala Z [68]	2021	Retrospective	134	NM	Unselected		13
Ceylan A [69]	2019	Cohort	82	EA	Positive	Epiduroscopy	12
Choi EJ [70]	2017	Retrospective	543	EA	Positive	MRI/Epidurography	6
Chun-Jing H [71]	2012	RCT	76	EA	Positive	Epidurography	6
Dagistan G [72]	2023	Retrospective	117	EA	Positive	MRI/Epidurography	6
De Andres J [73]	2017	RCT	55	NM	Unselected		12
Devulder J [74]	1999	RCT	40	EA	Positive	MRI/Epidurography	6
Ege F [75]	2024	Retrospective	72	EA	Positive	MRI/Epidurography	6
El Molla AF [76]	2016	RCT	42	EA	Positive	MRI/Epiduroscopy	6
Eldabe SS [77]	2019	RCT	116	NM	Unselected		9
Funao H [78]	2022	Retrospective	271	EA	Positive	Epiduroscopy/Epidurography	6
Garcia March G [79]	2015	Retrospective	119	NM	Positive	MRI	56
Gazzeri R [80]	2023	Retrospective	49	EA	Positive	Epidurography/Epiduroscopy	6
Gerdesmeyer L [81,82]	2013/2021	RCT	90	EA	Positive	CT/MRI/Epidurography	120
Gheith R [83]	2025	Retrospective	333	NM	Unselected		13
Goudman L [84]	2021	Cohort	259	NM	Unselected		12
Goudman L [85]	2020	Retrospective	119	NM	Unselected		19
Goudman L [86]	2024	Retrospective	11,934	NM	Unselected		84
Hagedorn JM [87]	2024	Retrospective	468	NM	Unselected		12
Hayek SM [88]	2015	Retrospective	203	NM	Unselected		44
Heavner JE [89]	1999	RCT	59	EA	Positive	Epidurography	12
Hossieni B [90]	2017	RCT	60	EA	Positive	MR/Epidurography	3
Joo EY [91]	2017	Retrospective	246	EA	Positive	MRI	6
Kaijankoski H [92]	2019	Retrospective	198	NM	Unselected		24
Kalagac FL [93]	2018	Cohort	54	EA	Positive	MRI	3
Kapural L [37,49]	2015/2016	RCT	198	NM	Unselected		24
Kapural L [94]	2020	Retrospective	105	NM	Unselected		12
Kim HJ [95]	2023	Retrospective	169	EA	Positive	CT/MRI/Epidurography	6
Kim JY [43]	2021	Retrospective	150	EA	Positive	MRI/Epidurography	3
Kim SB [96]	2012	RCT	43	EA	Positive	MRI/Epidurography	3
Kumar K [97,98]	2007/2008	RCT	100	NM	Unselected		6
Kumar K [99]	2006	Retrospective	220	NM	Positive	CT/MRI	98
La Grua M [59]	2023	Cohort	99	NM vs. EA	Positive	MRI	1
Labaran L [100]	2020	Retrospective	3104	NM	Unselected		36
Lee JH [101]	2014	Retrospective	114	EA	Positive	Epidurography	6
Lee SI [102]	2004	RCT	86	EA	Positive	MRI	6
Leong SL [103]	2021	RCT	100	NM	Unselected		3
Luikku AJ [104]	2025	Retrospective	256	NM	Unselected		109
Manchikanti L [32]	2003	RCT	39	EA	Positive	Epidurography	6
Manchikanti L [105]	2004	RCT	75	EA	Positive	CT/MR/Epidurography	12
Manchikanti L [106]	2005	RCT	83	EA	Positive	Epidurography	12
Manchikanti L [107,108]	2009/2012	RCT	120	EA	Positive	CT/MR/Epidurography	24
Manchikanti L [109]	1999	Retrospective	120	EA	Positive	Epidurography	12
Masopust V [110]	2021	Cohort	48	NM	Positive	MRI	36
Mekhail N [111]	2023	Retrospective	166	NM	Unselected		6
Mekhail NA [112]	2011	Retrospective	235	NM	Unselected		41
Metzger CS [113]	2020	Retrospective	420	NM	Unselected		12
Morozov IN [114]	2015	RCT	80	NM	Unselected		6
Muhammad S [115]	2017	Cohort	16	NM	Unselected		15
Nissen M [116]	2019	Retrospective	175	NM	Unselected		60
North J [60]	2020	RCT	140	NM	Unselected		3
North RB [117]	2005 a	RCT	50	NM	Unselected		6
North RB [118]	2005 b	RCT	24	NM	Unselected		34
North RB [119]	1991	Retrospective	50	NM	Positive	CT/Myelography	60
Oh Y [120]	2020	Retrospective	147	EA	Positive	MRI/Epidurography	6
Park CH [121]	2019	Retrospective	82	NM	Positive	Epidurography	12
Perez C [122]	2021	Cohort	85	NM	Unselected		24
Probst C [123]	1990	Retrospective	112	NM	Positive	CT/Myelogram	54
Puylaert M [124]	2023	Retrospective	191	NM	Unselected		127
Rapcan R [33]	2018	RCT	45	EA	Positive	MRI/Epiduroscopy	12
Rauck RL [125]	2023	Retrospective	652	NM	Unselected		36
Rigoard P [126]	2019	RCT	218	NM	Unselected		6
Rigoard P [127]	2021 a	RCT	100	NM	Unselected		12
Rigoard P [128]	2021 b	RCT	13	NM	Unselected		3
Rigoard P [129]	2022	Retrospective	108	NM	Unselected		12
Rufolo D [130]	2024	Retrospective	155	NM	Unselected		9
Russo M [131]	2016	Retrospective	256	NM	Unselected		6
Sanders RA [132]	2016	Retrospective	199	NM	Unselected		12
Sulaiman AI [133]	2021	Retrospective	50	EA	Positive	MRI/Epidurography	3
Taibi T [134]	2026	Retrospective	175	NM	Unselected		13
Takeshima N [135]	2009	Cohort	28	EA	Positive	Epiduroscopy	6
Tuijp SJ [136]	2018	Retrospective	35	EA	Positive	MRI/Epiduroscopy	6
Turner JA [137]	2010	Cohort	155	NM	Unselected		24
Van Buyten JP [138]	2001	Retrospective	217	NM	Unselected		40
van Gorp EJAA [51,139]	2016/2019	RCT	52	NM	Unselected		12
van Heteren EPZ [52]	2023	Cohort	75	NM	Unselected		12
Veihelmann A [140]	2006	RCT	99	EA	Positive	MRI/CT	12
Witkam RL [141]	2023	Retrospective	570	NM	Unselected		36
Yousef AAAM [31]	2010	RCT	38	EA	Positive	Epidurography	12

CT: Computed Tomography; MRI: Magnetic Resonance Imaging; n: Total Population Size.

**Table 3:** Summary of principal findings by fibrosis stratum.

Stratum	Studies	Patients n	Meta-Regression Network Heterogeneity		Highest-Ranked Node (Exploratory P-Score, Patients n)
PAIN Fibrosis-positive Fibrosis-unselected	16	1.270	−0.97	I^2^ = 89.49%	EA_HyalSal (0.986, n = 103)
	16	1.930	−0.63		SCS_PNFS (0.891, n = 102)
ODI Fibrosis-positive Fibrosis-unselected	10	915	−11.01	I^2^ = 89.40%	EA_Hyal (0.785, n = 79)
	13	1.286	−8.46		SCS_HF10 (0.839, n = 168)

The pain and ODI estimates shown are the subgroup coefficients from the mixed-effects meta-regression and are not directly comparable across strata, because the retained comparator structures differ between the subgroup networks and because fibrosis status is confounded with treatment family. Heterogeneity values are those of the corresponding networks. P-scores are exploratory ranking summaries and do not denote comparative superiority.

**Table 4:** Joint distribution of treatment family and fibrosis status across the included evidence.

Treatment Family	Fibrosis-Positive	Fibrosis-Unselected	Total
**All study families (N = 87)**			
Epidural adhesiolysis (EA) only	33	0	33
Neuromodulation (NM) only	6	47	53
Both EA and NM (single cohort)	1	0	1
Total	40	47	87
**Randomized controlled trials (N = 30)**			
Epidural adhesiolysis (EA)	16	0	16
Neuromodulation (NM)	0	14	14
Total	16	14	30

EA = epidural adhesiolysis; NM = neuromodulation. Counts are study families, derived from the master extraction dataset. The EA/fibrosis-unselected cell is empty in the full dataset and among randomized trials. Among randomized controlled trials, fibrosis status and treatment family are perfectly collinear: every fibrosis-positive trial evaluated EA and every fibrosis-unselected trial evaluated NM, so that no randomized trial delivered both families within one fibrosis stratum. The single study evaluating both families [59] was non-randomized and fibrosis-positive.

## Data Availability

The data presented in this study are available upon reasonable request from the corresponding author.

## Supplementary Materials

The following supporting information can be downloaded at: https://www.mdpi.com/article/10.3390/jcm15166480/s1, Supplementary File S1: PROSPERO-related amendments, search, study selection, and final dataset architecture; Supplementary File S2: Results [148].

## Abbreviations

The following abbreviations are used in this manuscript:

CMM	Conventional medical management
CT	Computed tomography
EA	Epidural adhesiolysis
FBSS	Failed back surgery syndrome
MRI	Magnetic resonance imaging
NM	Neuromodulation
ODI	Oswestry Disability Index
PSPS-T2	Persistent spinal pain syndrome type 2
SCS	Spinal cord stimulation
