# Supplementary material for: Structural and Functional Treatment of Persistent Spinal Pain Syndrome Type 2: A Fibrosis-Stratified Network Meta-Analysis of Epidural Adhesiolysis and Neuromodulation
Source: J Clin Med. 2026 Aug 21;15(16):6480. doi: 10.3390/jcm15166480 (PMC13514689; doi:10.3390/jcm15166480)
Supplement: Supplementary file 1 [file jcm-15-06480-s001.zip › jcm-4456722-supplementary.pdf]

Supplementary materials, datasets, figures, and tables

Contents

1. Supplementary File S1: PROSPERO-related amendments, search, study selection, and final dataset architecture 2

1.1. PROSPERO-related changes and the final architecture of the dataset. 2

1.2. Search strategy 3

2.Supplementary File S2. Results 4

2.1. Studies characteristics 4

2.2. Justification for Retention of Fibrosis-Positive Tier 3 Studies Below the Original PROSPERO n≥100 Threshold and 100%FBSS 4

2.3. Justification for Inclusion of Structurally Enriched Mixed-Population Adhesiolysis Trials 5

2.4. Justification for EA_HyperSal node assignment 6

2.5. Justification for Harmonization of Waddell disability scores 6

2.6. Justification for responder-threshold harmonization 7

2.7. Justification for inclusion of mixed chronic lumbar pain populations 7

2.8. Justification for inclusion of a prospective cohort study van Heteren et al. 2023 7

2.9. Network meta-analysis 8

2.10. Mixed-effects meta-regression 13

2.11. Inconsistency and Sensitivity Analyses 13

2.12. Quality Assessment 17

1. Supplementary File S1: PROSPERO-related amendments, search, study selection, and final dataset architecture

1.1 PROSPERO-related changes and the final architecture of the dataset.

This appendix documents the relationship between the original registered review framework and the final manuscript-specific analytic approach. It summarizes terminology modernization, study-level fibrosis-classification logic, comparator clarification, study-publication consolidation, tier architecture, and the post hoc exploratory emergence of the fibrosis-focused framework used in the present manuscript.

• Condition label: the manuscript uses PSPS-T2 as the preferred term while retaining FBSS as a historical search and literature term.

• Population stratification: the final manuscript uses fibrosis-positive versus studies without confirmed fibrosis to avoid falsely implying a clean fibrosis-negative comparator group.

• Primary analytic framework: the final program uses a frequentist random-effects netmeta approach because it proved more stable for repeated reruns, multi-arm handling, and control-anchored comparisons.

• Study/publication structure: the final analytic unit is the study family rather than each publication, preventing duplicate counting while preserving linked follow-up reports.

• Protocol-deviation sensitivity: small fibrosis-positive Tier 3 studies were not automatically excluded when analytically interpretable, but were flagged for grouped and/or leave-one-out sensitivity review.

Methodological note on fibrosis ascertainment.

Fibrosis-positive classification was intentionally multimodal and accepted contrast-enhanced MRI, epidurography, epiduroscopy, CT/myelography, or direct/intraoperative structural confirmation. This improved inclusiveness and reflected real-world evidence architecture, but it also introduces ascertainment heterogeneity because these modalities do not have identical sensitivity or biologic specificity. No robust modality-stratified effect analysis was performed in the present manuscript, and this should be regarded as a further source of interpretive uncertainty.

Handling of Multiple Publications from the Same Trial

For trials with multiple publications reporting outcomes at different follow-up timepoints (e.g., Gerdesmeyer 2013/2021, Kumar 2007/2008, Kapural 2015/2016, Rigoard 2019/2021), data were extracted according to the clinically retained timepoint and treated as linked reports from the same underlying study rather than as independent cohorts. Within-study correlations in multi-arm trials were preserved within the netmeta framework so that comparisons sharing a common control were not analyzed as independent observations. For the cumulative family-level analysis, repeated follow-up reports from the same underlying study were not treated as independent parallel studies; instead, linked publications were consolidated at study-family level, and the cumulative 3–24 month framework was interpreted as a retained primary pain window rather than as multiple unrelated observations. Time-stratified family reruns and representative time-window displays were then used as supportive analyses to clarify temporal behavior without duplicating the same cohort repeatedly in the main family-level interpretation. The studies by van Gorp (2016) and van Heteren (2023) were evaluated separately. Neither study utilized randomization, and they involved entirely different patient populations despite sharing the same study registration.

1.2. Search strategy

The full search strategy for all databases, including the specific Boolean operators and keywords, and filters used in title and abstract:

"failed back surgery syndrome" OR "post-laminectomy syndrome" OR "post laminectomy syndrome" OR "post-surgical spine pain" OR "pain after spinal surgery" OR "postoperative spinal pain" OR "chronic lumbosacral radiculopathy" OR "postoperative radiculopathy" OR "persistent spinal pain syndrome" OR "ICD-11" OR "postoperative epidural fibrosis" AND

"epidural adhesiolysis" OR "percutaneous adhesiolysis" OR "lysis of adhesions" OR "Racz procedure" OR "epidural neuroplasty" OR "neuroplasty" OR "adhesiolysis" OR "epidural lysis" OR "epidurolysis" OR "epiduroscopy" OR "trans-sacral canal plasty" OR "spinal endoscopy" OR "epiduroscopic lysis" OR "spinal cord stimulation" OR "dorsal root ganglion stimulation" OR "neuromodulation" OR "neurostimulation" OR "spinal stimulator" OR "high-frequency stimulation" OR "pulsed radiofrequency" NOT animals

2. Supplementary File S2. Results

2.1. Studies characteristics

Table S1: Characteristics and Network Structure by Fibrosis Status

Category	Total studies	Total patients	Fibrosis-positive studies	Fibrosis-positive patients	Without-confirmed-fibrosis studies	Without-confirmed-fibrosis patients
Overall database	87	33,504	40	4,180	47	29,324
Tier 1 randomized controlled trials	30	2,400	16	1,055	14	1,345
Tier 2 prospective cohorts	10	901	5	311	5	590
Tier 3 retrospective / registry studies	47	30,203	19	2,814	28	27,389

Legend. The final PSPS-T2 database comprised 87 studies and 33,504 patients, including 30 tier-1 randomized trials, 10 tier-2 prospective cohorts, and 47 tier-3 retrospective or registry studies. Across the full database, 40 studies were classified as fibrosis-positive and 47 as without confirmed fibrosis. Because the non-positive stratum was not uniformly established as fibrosis-negative, the preferred manuscript wording is fibrosis-positive versus without confirmed fibrosis.

2.2. Justification for Retention of Fibrosis-Positive Tier 3 Studies Below the Original PROSPERO n≥100 Threshold and 100%FBSS

The original PROSPERO-era threshold proposed exclusion of Tier 3 retrospective studies with fewer than 100 patients. During final dataset construction, this rule was not applied rigidly because it would have preferentially removed structurally selected fibrosis-positive studies from the adhesiolysis-relevant side of the evidence base rather than excluding a random subset of the literature. Accordingly, seven fibrosis-positive Tier 3 studies below the original n≥100 threshold were retained because they contributed clinically interpretable structural-fibrosis information, strengthened representation of the fibrosis-positive stratum, and informed the broader fibrosis-stratified evidence architecture. Their inclusion should therefore be understood as a data-driven and methodologically justified refinement of the original threshold rule rather than a post hoc lowering of standards.

Table S2. Justification for Inclusion of Fibrosis-Positive Tier 3 Studies

Study	Reason for retention despite original threshold	Justification
Tuijp_2018	Fibrosis-positive / structurally selected cohort	Retained because it directly informs the fibrosis-positive structural-adhesiolysis domain and would otherwise remove relevant fibrosis-confirmed evidence from the structurally dominant stratum.
Sulaiman_2021	Fibrosis-positive / structurally selected cohort	Retained because it contributes interpretable fibrosis-positive adhesiolysis data and helps preserve representation of clinically relevant structurally targeted treatment in the fibrosis-confirmed subgroup.
Park_2019	Fibrosis-positive / structurally selected cohort	Retained because it contributes procedure-relevant fibrosis-positive evidence and supports the structurally dominant side of the fibrosis-stratified framework.
North_1991	Fibrosis-positive / structurally selected cohort	Retained because it contributes historically important fibrosis-positive outcome information and helps prevent artificial depletion of the fibrosis-confirmed side of the comparative architecture.
Gazzeri_2023	Fibrosis-positive / structurally selected cohort	Retained because it provides interpretable structurally selected evidence relevant to fibrosis-positive treatment-response patterns.

Ege_2024	Fibrosis-positive / structurally selected cohort	Retained because it adds contemporary fibrosis-positive evidence and strengthens representation of the structurally confirmed adhesiolysis-relevant subgroup.
Joo_2017	Fibrosis-positive / structurally selected cohort	Retained because it informs the effect of hypertonic saline concentration within percutaneous EA, a core EA component used across the adhesiolysis literature and not exclusively postoperative.

Legend. These seven studies were retained despite the original PROSPERO-era Tier 3 n≥100 threshold because rigid exclusion would have disproportionately removed fibrosis-positive structurally selected studies rather than reducing bias evenly across the evidence base. Their inclusion was therefore considered methodologically justified within the final fibrosis-stratified master dataset and should be interpreted together with the study-handling and sensitivity framework.

Excluding these studies a priori would have preferentially weakened the fibrosis-positive structural-evidence side of the network and thereby introduced a new imbalance into the final comparative architecture.

2.3. Justification for Inclusion of Structurally Enriched Mixed-Population Adhesiolysis Trials

Several adhesiolysis studies, including Heavner et al. (1999), Veihelmann et al. (2006) and the Gerdesmeyer trial family (2013, 2021), enrolled mixed chronic postoperative/radicular pain populations rather than exclusively PSPS-T2-only cohorts. These studies were retained because the present manuscript evaluates fibrosis-associated treatment-response architecture rather than a narrowly surgery-exclusive epidemiologic subset.

Importantly, these trials did not represent nonspecific chronic low back pain populations. Instead, they enrolled clinically and structurally enriched radicular-pain cohorts characterized by epidural adhesions, failed conservative management, advanced degenerative pathology, postoperative changes, or procedure-relevant fibrosis-confirmation methods including MRI, CT, epidurography, and/or procedural adhesiolysis targeting.

The decision to retain these studies was based on four methodological considerations:

1. Structural relevance over diagnostic label alone

The biologic hypothesis of the present manuscript centers on structurally confirmed epidural fibrosis and adhesion-related pathology rather than exclusively on historical diagnostic terminology such as FBSS. Mixed cohorts with procedure-relevant fibrosis architecture remain mechanistically informative for the fibrosis-stratified framework.

2. Historical evolution of PSPS-T2/FBSS definitions

Earlier adhesiolysis literature frequently used heterogeneous terminology, including chronic lumbar radiculopathy, post-laminectomy syndrome, epidural fibrosis syndrome, or failed conservative therapy. Restricting inclusion only to studies using modern PSPS-T2 terminology would disproportionately exclude foundational structurally targeted adhesiolysis studies.

3. Avoidance of selective depletion of fibrosis-positive evidence

Rigid exclusion of structurally enriched mixed-population adhesiolysis trials would preferentially weaken the fibrosis-positive side of the comparative evidence architecture while leaving broad neuromodulation registries largely intact. This would introduce a new asymmetry into the final network structure.

4. Interpretation at study-family level rather than patient-level purity

The present analysis intentionally uses study-level fibrosis architecture rather than assuming biologically pure patient-level subgroup separation. Accordingly, these studies should be interpreted as contributing structurally enriched fibrosis-relevant evidence rather than as proving uniform fibrosis-positive status in every individual participant.

Accordingly, Heavner et al. (1999), Veihelmann et al. (2006) and Gerdesmeyer et al. (2013, 2021) were retained as structurally enriched fibrosis-relevant adhesiolysis studies within the exploratory fibrosis-stratified evidence framework. Their inclusion should therefore be interpreted as hypothesis-supportive rather than as definitive proof of a pure fibrosis-specific treatment effect.

Table S3. Justification for Inclusion of Structurally Enriched Fibrosis-Relevant Adhesiolysis Studies

Study	Population Characteristics	Structural Relevance	Reason for Retention
Veihelmann 2006	Chronic lumbar radicular pain including postoperative patients	MRI/CT-confirmed structural pathology; adhesiolysis-targeted intervention	Mechanistically relevant fibrosis-enriched cohort
Gerdesmeyer 2013/2021	Chronic lumbar radicular pain with disc pathology and failed back surgery components	CT/MRI/epidurography-guided adhesiolysis architecture	Structurally enriched mixed-population adhesiolysis trial family
Heavner 1999	Chronic radicular pain with epidurographic confirmation	Epidurography-confirmed adhesions	Procedure-relevant fibrosis architecture

2.4. Justification for EA_HyperSal node assignment

Studies were assigned to the EA_HyperSal node when the epidural adhesiolysis protocol included a deliberate hyperosmolar or high-volume saline component intended to enhance epidural spread, disrupt adhesions, dilute inflammatory mediators, or augment mechanical/chemical adhesiolysis. This included protocols using 3% or 5% hypertonic saline, as well as studies using large-volume normal saline irrigation, typically 60–80 mL, when the injected volume was explicitly used as part of the adhesiolysis strategy.

Although 10% hypertonic saline has historically been used in some adhesiolysis protocols, the biologic and procedural rationale of lower-concentration hypertonic saline remains aligned with the same treatment family: chemically assisted epidural adhesiolysis through an osmotic saline component. Similarly, high-volume normal saline protocols may partly compensate for lower osmolarity by increasing hydrodissection and epidural lavage effects. Therefore, these studies were grouped under EA_HyperSal as a pragmatic treatment-family node rather than as a strictly concentration-specific node.

This classification was chosen to preserve clinical interpretability and network connectivity while avoiding over-fragmentation of sparse treatment nodes. Because differences in saline concentration and volume may introduce within-node heterogeneity, these studies were reviewed carefully in sensitivity and narrative analyses, and the EA_HyperSal node should be interpreted as a family-level estimate rather than evidence for one specific saline concentration or volume.

2.5. Justification for Harmonization of Waddell disability scores

When nonstandard functional disability measures were reported, harmonized directional transformations were performed where methodologically justifiable to permit inclusion in pooled comparative analyses. For example, El Molla et al. reported functional outcomes using the Waddell disability index rather than the Oswestry Disability Index (ODI) used by most included studies. To preserve network connectivity and avoid exclusion of otherwise eligible data, Waddell disability scores were directionally harmonized into standardized functional outcome estimates suitable for pooled comparative analysis.

Because both instruments assess pain-related functional impairment in chronic lumbar spine disorders, the transformation was performed at the level of relative functional improvement rather than assuming direct numerical equivalence between scales.

The conversion was intended to preserve treatment-response directionality and approximate relative magnitude for network synthesis rather than to imply strict psychometric interchangeability between

the Waddell disability index and ODI. Accordingly, transformed values should be interpreted as harmonized disability estimates rather than true ODI measurements.

Similar pragmatic harmonization approaches between related outcome instruments are recognized in evidence synthesis methodology, particularly when necessary to preserve clinically relevant comparative data and network structure.[1] Because only a limited number of studies required such transformation, the overall influence on pooled network estimates was considered modest; however, this methodological step remains a potential source of uncertainty and heterogeneity and was interpreted cautiously during narrative synthesis.

[1] Higgins, J.P.T.; Li, T.; Deeks, J.J. Chapter 6: Choosing effect measures and computing estimates of effect. In *Cochrane Handbook for Systematic Reviews of Interventions*; Higgins, J.P.T., Thomas, J., Chandler, J., Cumpston, M., Li, T., Page, M.J., Welch, V.A., Eds.; Version 6.4. Cochrane; 2023. Available online: https://www.cochrane.org/authors/handbooks-and-manuals/handbook/archive/v6.4 (accessed on 1 August 2026).

2.6. Justification for responder-threshold harmonization

Most included studies defined responder outcomes using a ≥50% pain reduction threshold. However, a small number of studies reported responder rates using a ≥30% pain improvement criterion only. These studies were retained because a 30% reduction in pain intensity is widely recognized as representing clinically meaningful improvement in chronic pain research and is accepted in IMMPACT recommendations and multiple interventional pain trials.

Excluding these studies would have resulted in unnecessary loss of data and reduced network connectivity, particularly within sparsely populated treatment nodes. Because responder thresholds were not fully uniform across the literature, responder analyses were interpreted as reflecting broadly clinically meaningful treatment response rather than a perfectly identical binary endpoint across all studies.

Studies using ≥30% thresholds represented only a small minority of the dataset, and the primary interpretation of responder outcomes focused on relative treatment-pattern consistency rather than absolute pooled response rates. Nonetheless, variation in responder definitions may have contributed modestly to heterogeneity within responder analyses.

2.7. Justification for inclusion of mixed chronic lumbar pain populations

Several included studies enrolled mixed chronic lumbar pain populations that were not exclusively limited to PSPS-T2/FBSS patients. These studies were retained when postoperative patients constituted a clinically meaningful subgroup and when the intervention mechanism remained directly relevant to postoperative epidural fibrosis, adhesions, radicular pain, or related postoperative pain generators.

Exclusion of such studies would have substantially reduced available evidence and weakened network connectivity, particularly for older adhesiolysis literature in which postoperative and non-postoperative radicular pain populations were frequently analyzed together.Importantly, the biologic rationale underlying adhesiolysis and fibrosis-targeted interventions remained applicable across these cohorts because epidural scarring, adhesions, inflammation, and restricted epidural spread are mechanistically shared phenomena.

Nevertheless, inclusion of mixed populations may have diluted postoperative-specific treatment estimates and contributed to clinical heterogeneity. These studies were therefore interpreted cautiously and considered supportive rather than definitive evidence for PSPS-T2-specific treatment effects.

2.8. Justification for inclusion of a prospective cohort study van Heteren et al. 2023

A randomized trial (van Gorp et al., 2016, 2019; n = 52) and a prospective cohort study (van Heteren et al., 2023; n = 75) share a study registration but evaluate different populations. Consequently, the van Heteren et al. dataset was kept in the primary evidence base and risk-of-bias assessment, but included in the sensitivity analyses of the network meta-analyses (Section 2.11).

2.9. Network meta-analysis

Table S4. The evidence network of trials included (Network Meta Analysis of Pain Intensity Score, ODI and Responder): 15 RCTs, 3 cohort studies, and 1 retrospective trials.

Tier 1 Baseline Demographics NMA

Study_ID	Category_ID	Treatment_Node	N_BL	Outcome
Bolash_2019	2	SCS_HF10	50	
Bolash_2019	2	SCS_LF	49	Pain, ODI, Responder
ChunJing_2012	1	EA_HyperSal	38	
ChunJing_2012	3	EA_Std	38	Pain, Responder
DeAndres_2017	2	SCS_HF10	26	
DeAndres_2017	2	SCS_LF	29	Pain, ODI, Responder
Devulder_1999	1	EA_Hyal	20	
Devulder_1999	3	Control	20	Responder
ElMolla_2016	1	EA_Mechanical	20	
ElMolla_2016	1	EA_HyalSal	22	Pain, ODI
Eldabe_2019	2	SCS_PNFS	56	
Eldabe_2019	3	Control	60	Pain, Responder
Gerdesmeyer_2013_2021	1	EA_HyalSal	46	
Gerdesmeyer_2013_2021	3	Control	44	Pain, ODI, Responder
Heavner_1999	1	EA_HyalSal	17	
Heavner_1999	1	EA_HyperSal	15	
Heavner_1999	3	Control	17	
Heavner_1999	1	EA_Hyal	10	Pain, Responder
Kim_2012	1	EA_Hyal	23	
Kim_2012	3	EA_Std	20	Pain, ODI, Responder
Kumar_2007_2008	2	SCS_LF	52	
Kumar_2007_2008	3	Control	48	Pain, ODI, Responder
Manchikanti_2003	1	EA_Mechanical	23	
Manchikanti_2003	3	EA_Std	16	Pain, ODI, Responder
Manchikanti_2004	1	EA_HyperSal	25	
Manchikanti_2004	1	EA_Mechanical	25	
Manchikanti_2004	3	EA_Std	25	Pain, ODI, Responder
Manchikanti_2005	1	EA_Mechanical	50	
Manchikanti_2005	3	EA_Std	33	Pain, ODI, Responder
Manchikanti_2009_2012	1	EA_HyperSal	60	
Manchikanti_2009_2012	3	EA_Std	60	Pain, ODI, Responder
Morozov_2015	2	SCS_LF	45	
Morozov_2015	3	CMM	35	Pain, ODI, Responder
North_2005_a	2	SCS_LF	24	
North_2005_a	3	ReOp	26	Responder
North _2020	2	SCS_SUBP	70	
North_2020	2	SCS_LF	70	Responder
Yousef_2010	1	EA_HyperSal	20	
Yousef_2010	1	EA_HyalSal	18	Pain
Kapural_2015_2016	2	SCS_HF10	92	
Kapural_2015_2016	2	SCS_LF	87	Pain, ODI, Responder

Rapcan_2018	1	EA_Mechanical	22	
Rapcan_2018	1	EA_Hyal	23	Pain, ODI
Rigoard_2019	2	SCS_LF	110	
Rigoard_2019	3	CMM	108	Pain, Responder
Rigoard_2021_CUMPNS	2	SCS_PNFS	6	
Rigoard_2021_CUMPNS	2	SCS_LF	7	Pain, ODI
Leong_2021	2	SCS_LF	45	
Leong_2021	2	SCS_Burst	55	Pain
vanGorp_2016_2019	2	SCS_PNFS	28	
vanGorp_2016_2019	2	SCS_LF	24	Pain, ODI, Responder
Veihelmann_2006	1	EA_HyperSal	47	
Veihelmann_2006	3	CMM	52	Pain, ODI
Lee_2004	1	EA_HyperSal	41	
Lee_2004	1	EA_Mechanical	45	Pain
**Tier 2 Baseline Demographics NMA**	**Category_ID**	**Treatment_Node**	**N_BL**	**Outcome**
Muhammad_2017	2	SCS_HF10	8	
Muhammad_2017	2	SCS_Burst	8	Pain
Perez_2021	2	SCS_All	39	
Perez_2021	3	Control	46	Pain, ODI, Responder
Kalagac_2018	1	EA_Hyal	33	
Kalagac_2018	3	Control	21	Pain, Responder
vanHeteren_2023	2	SCS_PNFS	54	
vanHeteren_2023	2	SCS_LF	21	Pain, ODI
LaGrua_2023	2	DRG	75	
LaGrua_2023	1	EA_Hyal	24	Pain
Turner_2010	2	SCS_LF	51	
Turner_2010	3	Control	39	Pain, ODI, Responder
**Tier 3 Baseline Demographics NMA**	**Category_ID**	**Treatment_Node**	**N_BL**	**Outcome**
Bretherton_2022	2	SCS_HF10	275	
Bretherton_2022	2	SCS_Burst	99	Pain, Responder
Joo_2017	1	EA_HyperSal	93	
Joo_2017	3	EA_Std	153	Pain, ODI, Responder
Lee_2014	1	EA_Mechanical	52	
Lee_2014	3	EA_Std	62	Responder
Manchikanti_1999	1	EA_HyperSal	60	
Manchikanti_1999	1	EA_Mechanical	60	Responder
Mekhail_2023	2	SCS_All	166	
Mekhail_2023	3	Control	218	Pain, ODI, Responder

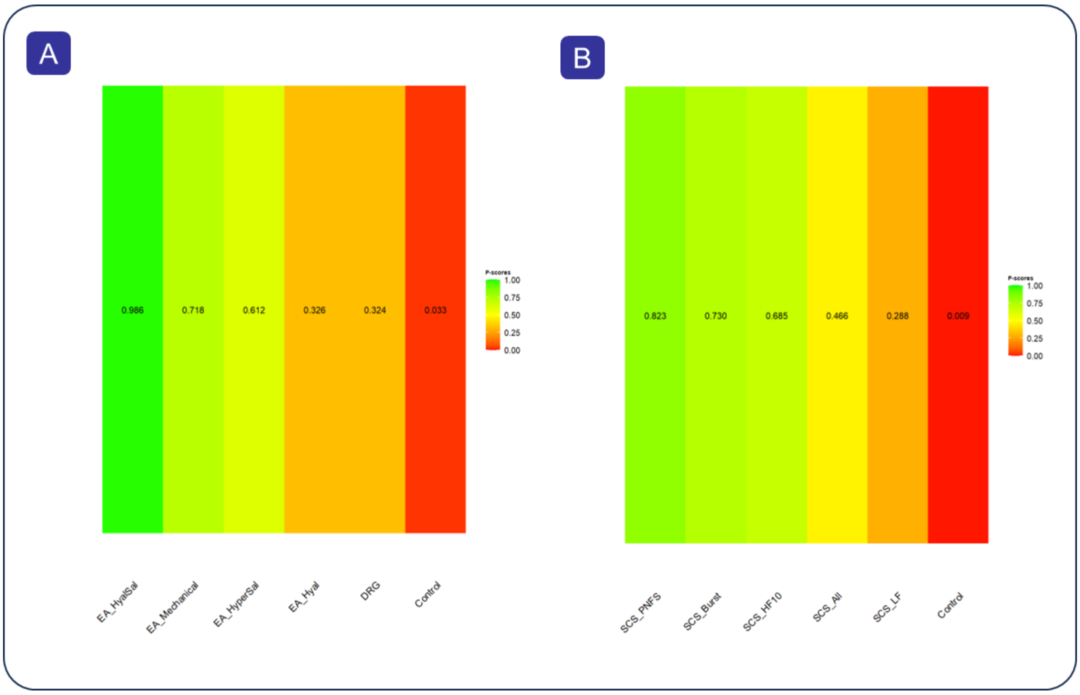

Figure S1. P-score Ranking of the Pooled Pain Network. Random-effects frequentist pain network ranking across all eligible pooled studies. Higher P-scores indicate better relative ranking. The pooled pain network ranked EA_HyalSal highest, followed by SCS_PNFS, EA_Mechanical, SCS_Burst, and SCS_HF10.

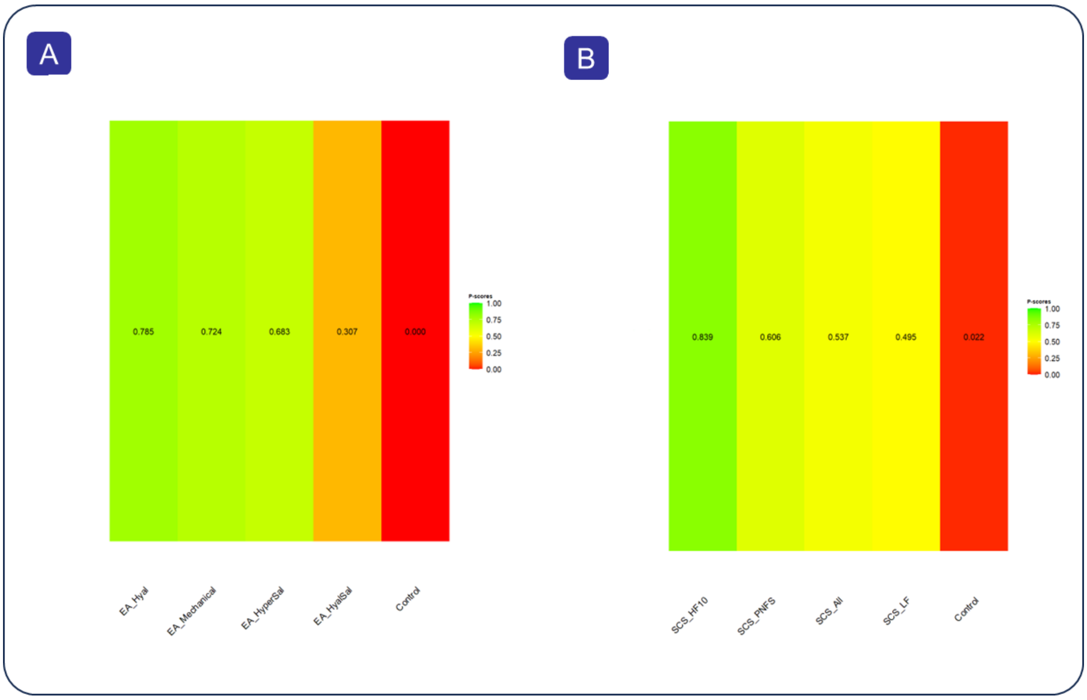

Figure S2. P-score Ranking of the Pooled ODI Network. P-score Ranking of the Supportive ODI Network Supportive ODI random-effects ranking using EA_Std as reference. Higher P-scores indicate better relative ranking. EA_HyperSal ranked highest, followed by EA_Hyal, EA_Mechanical, SCS_HF10, and SCS_PNFS.

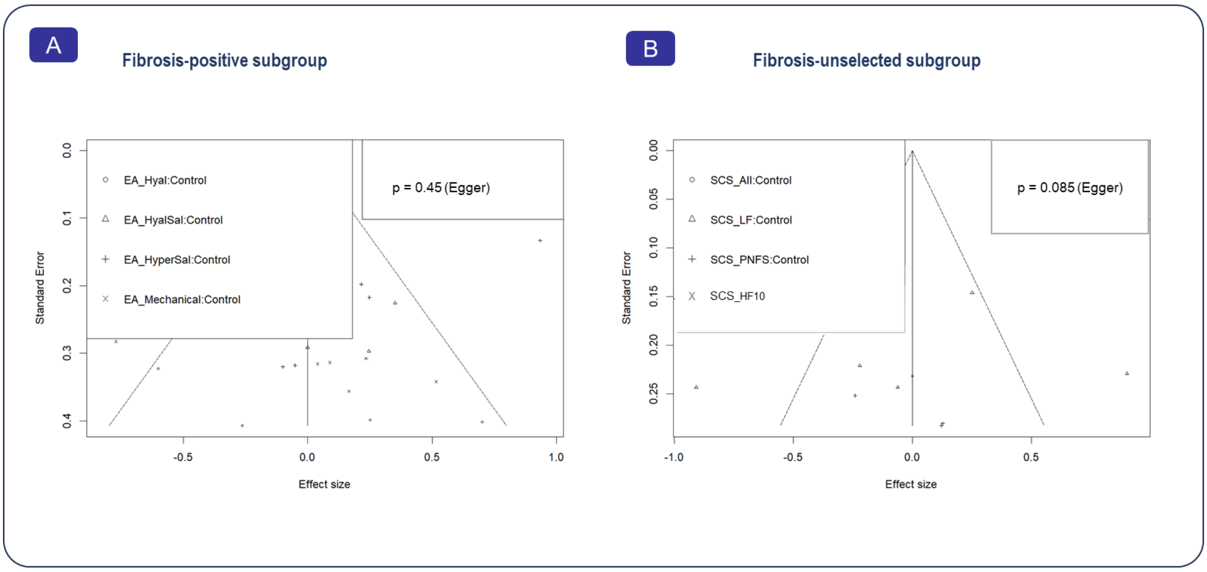

Figure S3. Pain - Funnel plot (A. positive and B. unselected)

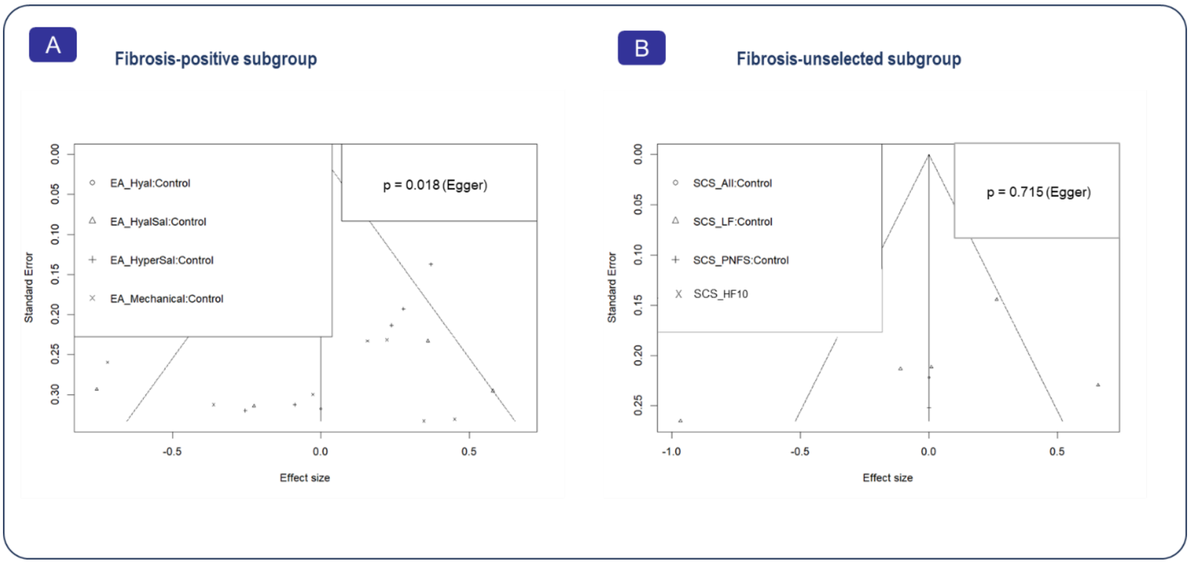

Figure S4. ODI - Funnel plot (positive and unselected)

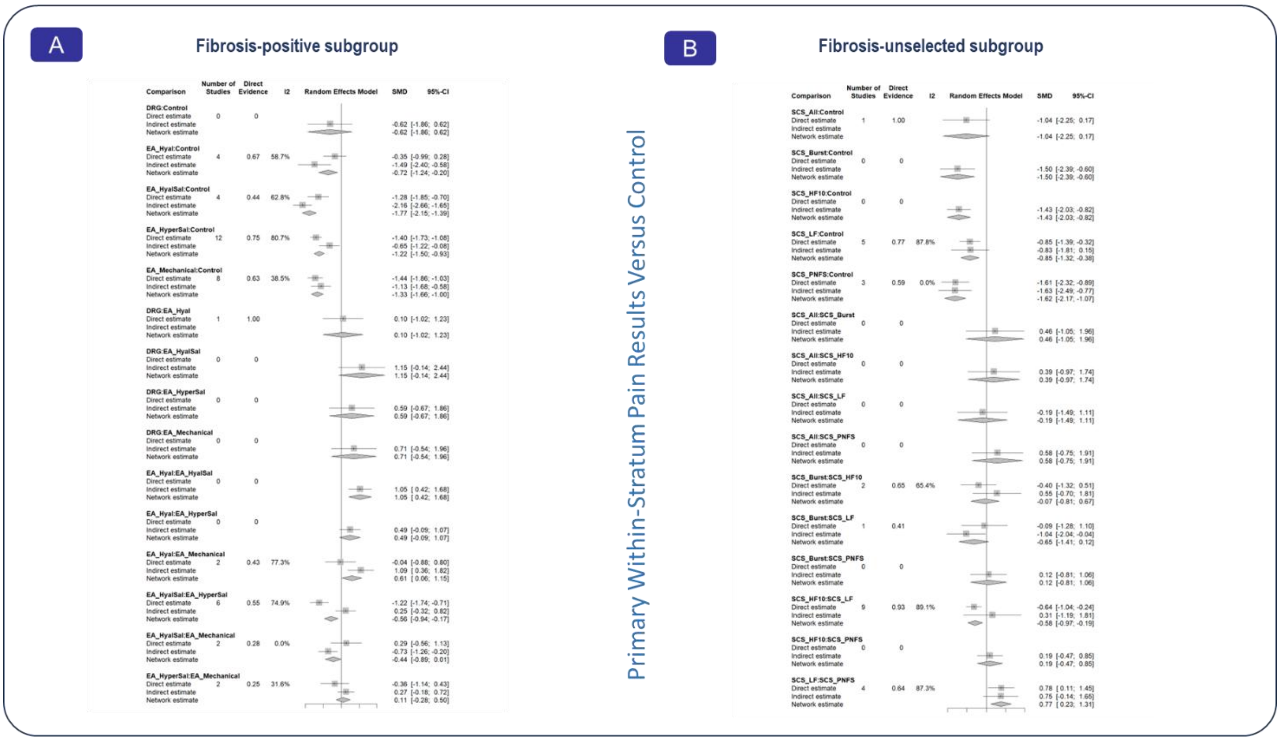

Figure S5. Netsplitt Pain

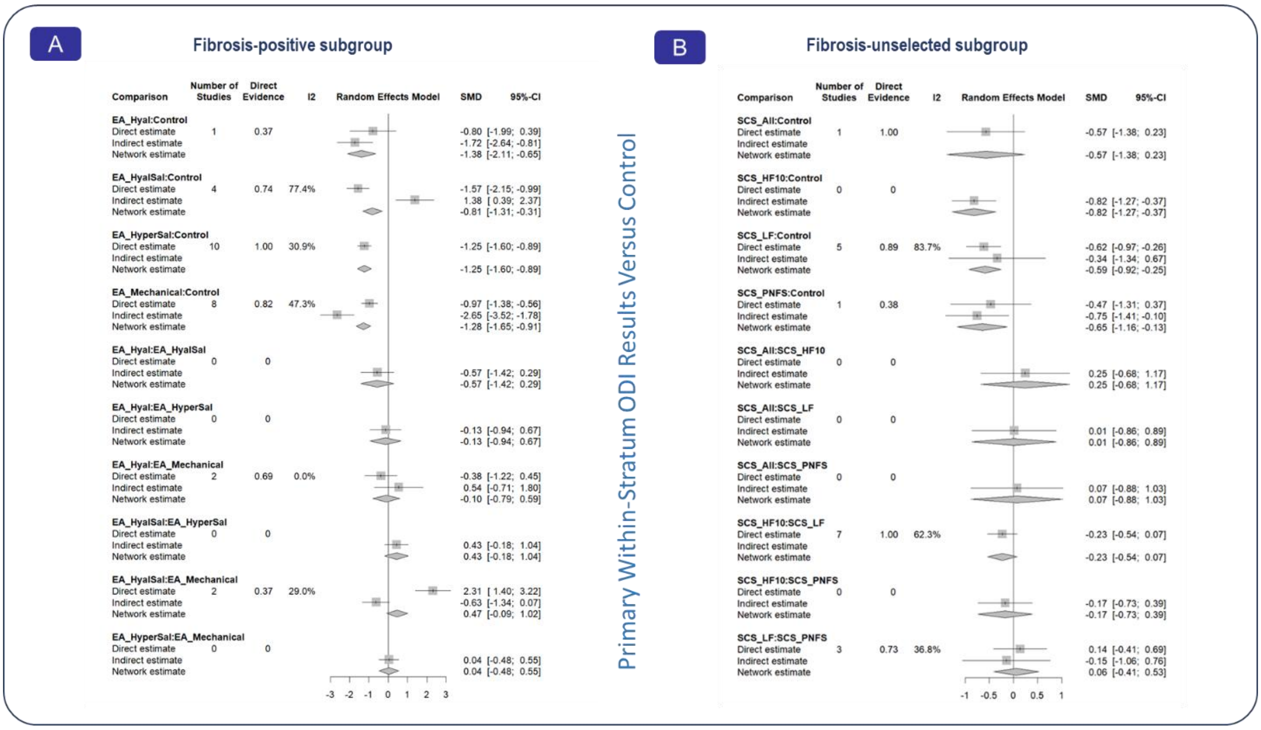

Figure S6. Netsplit ODI

2.10. Mixed-effects meta-regression

The mixed-effects meta-regression demonstrated substantial residual heterogeneity ($I^2 = 76.72\%$), indicating that a considerable proportion of variability across studies remained unexplained after accounting for the subgroup moderator. The residual heterogeneity test was statistically significant (QE = 188.98, $p < 0.0001$), confirming persistent between-study differences. However, the moderator analysis was also highly significant (QM = 104.37, $p < 0.0001$), suggesting that subgroup classification significantly influenced responder outcomes. Specifically, the "Positive" subgroup showed a strong and statistically significant association with responder outcome (estimate = 2.3608, 95% CI: 1.8435 to 2.8782, $p < 0.0001$), indicating a markedly higher likelihood of response compared with the reference category. Similarly, the "Unselected" subgroup was also significantly associated with improved responder outcome (estimate = 1.4233, 95% CI: 0.8584 to 1.9883, $p < 0.0001$), although the magnitude of effect was smaller than that observed in the Positive subgroup. Since van Heteren et al. 2023 never entered any meta-regression (no control arm), that reproduction validates the whole pipeline. Conclusions are unaffected.

2.11. Inconsistency and Sensitivity Analyses

TIER1 (PAIN)

In this tier 1 analysis of pain outcomes, most active interventions demonstrated statistically significant reductions in pain compared with control, as reflected by negative SMDs. The largest effects were observed with EA_HyalSal (SMD −1.80), SCS_PNFS (SMD−1.83), EA_HyperSal (SMD−1.28), and SCS_HF10 (SMD−1.57), all with highly significant p-values (<0.0001), indicating strong evidence of benefit. EA_Mechanical (SMD−1.34), EA_Hyal (SMD−0.73), and SCS_LF (SMD−0.92) also showed significant pain reduction, though of relatively smaller magnitude. In contrast, SCS_Burst (SMD−1.00) did not reach statistical significance ($p = 0.0839$), suggesting uncertainty in its effect despite a seemingly large point estimate. However, the analysis is characterized by substantial heterogeneity ($I^2 = 78.1\%$) and significant inconsistency between designs ($p < 0.0001$), indicating that variability across studies and disagreement between direct and indirect evidence may limit confidence in the relative treatment estimates.

The netsplit analysis showed that most interventions were associated with lower pain scores compared with the control group, although the magnitude and consistency of effects varied across comparisons. Significant network estimates favoring the interventions over control were observed for EA_HyalSal (SMD = −1.80, 95% CI: −2.16 to −1.43), EA_HyperSal (SMD = −1.28, 95% CI: −1.56 to −1.00), EA_Mechanical (SMD = −1.34, 95% CI: −1.66 to −1.02), SCS_LF (SMD = −0.92, 95% CI: −1.38 to −0.46), SCS_PNFS (SMD = −1.83, 95% CI: −2.35 to −1.32), and EA_Hyal (SMD = −0.73, 95% CI: −1.23 to −0.23). Comparisons between active interventions revealed that EA_Hyal was significantly less effective than EA_Mechanical (SMD = 0.61, 95% CI: 0.08 to 1.13), while EA_HyalSal showed significantly better pain reduction than EA_HyperSal (SMD = −0.52, 95% CI: −0.89 to −0.15) and EA_Mechanical (SMD = −0.45, 95% CI: −0.88 to −0.03). SCS_LF also appeared less effective than SCS_PNFS (SMD = 0.92, 95% CI: 0.37 to 1.46). In contrast, the comparison between EA_HyperSal and EA_Mechanical was not statistically significant, as the confidence interval crossed zero. Several comparisons demonstrated notable heterogeneity, particularly EA_Hyal versus Control, EA_HyalSal versus Control, and SCS_LF versus Control, indicating variability across included studies.

The network ranking analysis for pain outcomes in Tier 1 studies demonstrated notable differences in the probability of treatment effectiveness across interventions. SCS_PNFS achieved the highest P-score (0.895), indicating the greatest likelihood of being the most effective treatment for pain reduction, closely followed by EA_HyalSal (0.885). SCS_HF10 also ranked favorably with a high P-score of 0.731, suggesting strong comparative efficacy. Moderate rankings were observed for EA_Mechanical (0.575) and EA_HyperSal (0.518), indicating intermediate effectiveness. In contrast, SCS_Burst (0.392), SCS_LF (0.287), and EA_Hyal (0.211) showed lower probabilities of superior pain relief relative to the higher-ranked interventions. Control had the lowest P-score (0.005),

confirming that active interventions overall were substantially more effective than no treatment or standard care. Overall, the ranking suggests that neuromodulation approaches, particularly SCS_PNFS and SCS_HF10, together with EA_HyalSal, may provide the most favorable pain outcomes among Tier 1 studies.

TIER1 + TIER2 (PAIN)

In this tier 1+2 network meta-analysis of pain outcomes, most interventions showed meaningful reductions in pain compared with control, although the strength of evidence varied. The largest and most consistent effects were observed with EA_HyalSal (SMD −1.80), SCS_PNFS (SMD−1.61), SCS_HF10 (SMD−1.44), EA_Mechanical (SMD−1.34), and EA_HyperSal (SMD−1.27), all of which were highly statistically significant ($p < 0.0001$), indicating robust efficacy. EA_Hyal (SMD−0.73) and SCS_LF (SMD−0.85) also demonstrated significant but comparatively moderate effects. SCS_Burst showed a significant reduction (SMD−1.48, $p = 0.0035$), whereas SCS_All (SMD−1.04, $p = 0.0707$) and DRG (SMD−0.63, $p = 0.3235$) did not reach statistical significance, suggesting uncertainty around their effectiveness despite favorable point estimates. Importantly, the analysis revealed substantial heterogeneity ($I^2 = 80.4\%$) and significant inconsistency between study designs ($p < 0.0001$), indicating considerable variability and disagreement between direct and indirect comparisons.

After exclusion of the van Heteren et al. 2023 cohort in the sensitivity analyses, network heterogeneity remained substantial ($I^2 = 88.3\%$), with significant within-design heterogeneity ($Q = 74.52$, 8 df, $p < 0.001$) and between-design inconsistency ($Q = 11.19$, 2 df, $p = 0.004$) relative to the primary analysis. Nevertheless, removing the cohort eliminated significant local inconsistency during node-splitting (all $p \geq 0.32$). Node-splitting p-values of ≥ 0.32 mean that direct and indirect evidence are now in harmony. The loops are consistent, making the final relative effect estimates much more reliable.

TIER1(ODI)

This analysis shows that all evaluated interventions significantly improve disability compared with control, as all SMDs are negative and their 95% confidence intervals do not cross zero. The largest reductions in disability are observed with EA_Hyal (SMD −1.38), EA_Mechanical (SMD−1.27), and EA_HyperSal (SMD−1.27), indicating substantial clinical benefit. Moderate but still significant effects are seen with SCS_HF10 (SMD−0.93), EA_HyalSal (SMD−0.83), SCS_PNFS (SMD−0.81), and SCS_LF (SMD−0.71). This suggests that both EA-based and SCS-based interventions are effective in reducing disability.However, the analysis demonstrates considerable heterogeneity ($I^2 = 76.5\%$), indicating substantial variability across studies. In addition, both within-design and between-design inconsistency tests are statistically significant ($p < 0.0001$), suggesting disagreement between direct and indirect evidence in the network.

The node-splitting analysis demonstrated that most interventions were associated with improved disability scores compared with control, although the consistency between direct and indirect evidence varied across comparisons. Network estimates showed significant reductions in ODI favoring EA_Hyal (SMD: −1.38, 95% CI: −2.03 to −0.72), EA_HyalSal (SMD: −0.83, 95% CI: −1.28 to −0.38), EA_Mechanical (SMD: −1.27, 95% CI: −1.61 to −0.94), SCS_LF (SMD: −0.71, 95% CI: −1.16 to −0.26), and SCS_PNFS (SMD: −0.81, 95% CI: −1.44 to −0.17) versus control, indicating clinically favorable effects on disability reduction. However, inconsistency between direct and indirect evidence was observed in some comparisons, particularly EA_HyalSal versus Control and EA_HyalSal versus EA_Mechanical, where the direct and indirect estimates differed substantially in both magnitude and direction. Heterogeneity ranged from low to substantial, with I^2 values of 0% for EA_Hyal versus EA_Mechanical and SCS_LF versus SCS_PNFS, but exceeded 75% for EA_HyalSal versus Control and SCS_LF versus Control, suggesting variability among included studies. Comparisons between active interventions, including EA_Hyal versus EA_Mechanical and SCS_LF versus SCS_PNFS, showed no statistically significant differences in the network estimates, indicating

relatively comparable effectiveness among these treatment approaches for ODI improvement.The netrank shows EA_Hyal (P-score = 0.833) ranks highest, indicating the greatest probability of being the most effective intervention for reducing disability, followed closely by EA_Mechanical (0.793) and EA_HyperSal (0.790), suggesting that EA-based therapies dominate the top ranks. SCS_HF10 (0.516) occupies a middle position with moderate effectiveness. Lower rankings are observed for SCS_PNFS (0.394) and EA_HyalSal (0.394), indicating comparatively weaker performance, while SCS_LF (0.278) ranks near the bottom among active treatments. As expected, Control (0.001) is the least effective. Overall, the ranking suggests that EA family are more likely to provide superior improvements in ODI compared to SCS family, although these rankings should be interpreted cautiously given the previously observed heterogeneity and inconsistency in the network.

TIER2(ODI)

This analysis shows limited and less consistent evidence of benefit compared with Tier 1 results. Only the pooled SCS_All category demonstrates a statistically significant reduction in disability versus control (SMD −0.57, 95% CI −1.01 to −0.14; p = 0.0097), indicating a modest but meaningful improvement. In contrast, when disaggregated, SCS_LF (SMD 0.04, p = 0.8615) and SCS_PNFS (SMD 0.28, p = 0.4150) show no significant effect, with confidence intervals crossing zero and point estimates close to or even favoring control. This suggests that the apparent benefit in the combined SCS group may be driven by aggregation rather than consistent effects across individual modalities.Importantly, heterogeneity and inconsistency could not be estimated (tau^2 and I^2 not available), and all Q statistics are zero. This typically reflects a very sparse network with insufficient comparisons or lack of closed loops, meaning that no indirect evidence is available to assess consistency.

The node-splitting analysis showed generally limited evidence with few direct comparisons, but some treatments demonstrated favorable effects over control. SCS_All significantly reduced ODI scores compared with control in the network estimate (SMD: −0.57, 95% CI: −1.01 to −0.14), suggesting improved disability outcomes. In contrast, SCS_LF versus control (SMD: 0.04, 95% CI: −0.41 to 0.49) and SCS_PNFS versus control (SMD: 0.28, 95% CI: −0.40 to 0.96) were not statistically significant, indicating no clear advantage over control. Comparisons between active interventions were largely based on indirect evidence alone, reflecting the sparse network structure. SCS_All showed a significantly greater reduction in ODI compared with SCS_PNFS (SMD: −0.86, 95% CI: −1.66 to −0.05), while the comparison with SCS_LF approached significance but crossed the null value (SMD: −0.61, 95% CI: −1.24 to 0.01). Similarly, SCS_LF and SCS_PNFS did not differ significantly from one another (SMD: −0.24, 95% CI: −0.75 to 0.26). This netrank shows a markedly simplified and less robust ranking pattern compared to Tier 1. SCS_All (P-score = 0.983) overwhelmingly ranks as the most effective intervention, suggesting a very high probability of being the best treatment for reducing ODI within this limited network. In contrast, Control (0.456) and SCS_LF (0.428) occupy intermediate and relatively similar positions, indicating little distinction between them in terms of effectiveness. Notably, SCS_PNFS (0.134) ranks lowest, suggesting it is the least effective among the evaluated treatments.

TIER1 + TIER2(ODI)

In this tier 1+2 network meta-analysis of ODI outcomes, several interventions demonstrated statistically significant improvements in disability compared with control, although effect sizes were generally more modest than those observed for pain. The largest and most consistent reductions in ODI were seen with EA_Hyal (SMD −1.38), EA_Mechanical (SMD−1.27), and EA_HyperSal (SMD−1.27), all with strong statistical significance ($p < 0.0001$), indicating substantial functional

benefit. EA_HyalSal (SMD−0.83) and SCS_HF10 (SMD−0.82) also showed significant improvements, while SCS_LF (SMD−0.60) and SCS_PNFS (SMD−0.64) demonstrated smaller but still statistically significant effects. In contrast, SCS_All (SMD−0.57) did not reach statistical significance ($p = 0.2495$), suggesting uncertainty in its overall effectiveness. Despite these favorable findings, the analysis is marked by considerable heterogeneity ($I^2 = 76.5\%$) and significant inconsistency between study designs ($p < 0.0001$), indicating variability in study results and potential disagreement between direct and indirect evidence. Excluding van Heteren et al. (2023) in a sensitivity analysis did not alter the findings. Since this cohort lacked a control-arm contrast, the direction and statistical significance of every network meta-analysis conclusion remained unchanged. In summary, EA-based interventions appear to provide the greatest improvement in ODI, with some SCS modalities also beneficial.

<u>Comparison</u>

For pain outcomes, the Tier 1 and Tier 1+2 sensitivity analyses were largely consistent with the all-tier network meta-analysis, reinforcing the robustness of the main findings. Across all analyses, EA_HyalSal, SCS_PNFS, SCS_HF10, EA_Mechanical, and EA_HyperSal consistently demonstrated the largest and most statistically significant reductions in pain, indicating stable treatment effects irrespective of study quality inclusion criteria. The magnitude of effect estimates remained very similar between analyses, suggesting that inclusion of lower-tier studies did not substantially alter the direction or strength of the evidence. However, some differences were observed for SCS_Burst and SCS_All. In the all-tier analysis, SCS_Burst showed a significant benefit, whereas in the Tier 1 analysis it lost statistical significance despite a large effect estimate, likely reflecting reduced precision due to fewer high-quality studies. The Tier 1+2 analysis restored significance for SCS_Burst, suggesting that moderate-quality studies contributed additional evidence supporting its efficacy. Similarly, DRG and SCS_All remained non-significant in both the all-tier and Tier 1+2 analyses, indicating persistent uncertainty regarding their effectiveness. Importantly, heterogeneity and inconsistency remained high across all analyses (I^2 ranging from 22.5% to 92.4%, $p < 0.0001$), demonstrating that variability between studies and disagreement between direct and indirect evidence persisted even after restricting analyses to higher-quality studies. In summary, the sensitivity analyses confirm the robustness of the primary pain findings, particularly for EA-based interventions and certain SCS modalities.

For ODI outcomes, the Tier 1 and Tier 1+2 analyses also showed strong agreement with the all-tier analysis, supporting the consistency of the disability-related findings. EA_Hyal, EA_Mechanical, and EA_HyperSal consistently produced the largest improvements in ODI across all analyses, indicating that EA-based interventions provide the most substantial functional benefit. Moderate but significant improvements with EA_HyalSal, SCS_HF10, SCS_LF, and SCS_PNFS were also maintained across the all-tier, Tier 1, and Tier 1+2 analyses, suggesting that these findings are robust to study quality restrictions. A notable discrepancy emerged with SCS_All: it was non-significant in the all-tier and Tier 1+2 analyses but became statistically significant in the isolated Tier 2 analysis. However, the Tier 2 network was sparse, lacked closed loops, and could not estimate heterogeneity or inconsistency, indicating that this isolated finding should be interpreted cautiously and may reflect instability due to limited evidence. Compared with the pain outcome, ODI effect sizes were generally smaller, although the relative ranking of interventions remained similar. Additionally, heterogeneity and inconsistency remained substantial and statistically significant in the all-tier, Tier 1, and Tier 1+2 analyses (I^2 approximately 76%), suggesting persistent variability across studies that was not fully explained by study quality. In conclusion, the sensitivity analyses support the reliability of the main ODI findings, particularly the superior and consistent benefit of EA-based interventions on disability reduction.

2.12. Quality Assessment

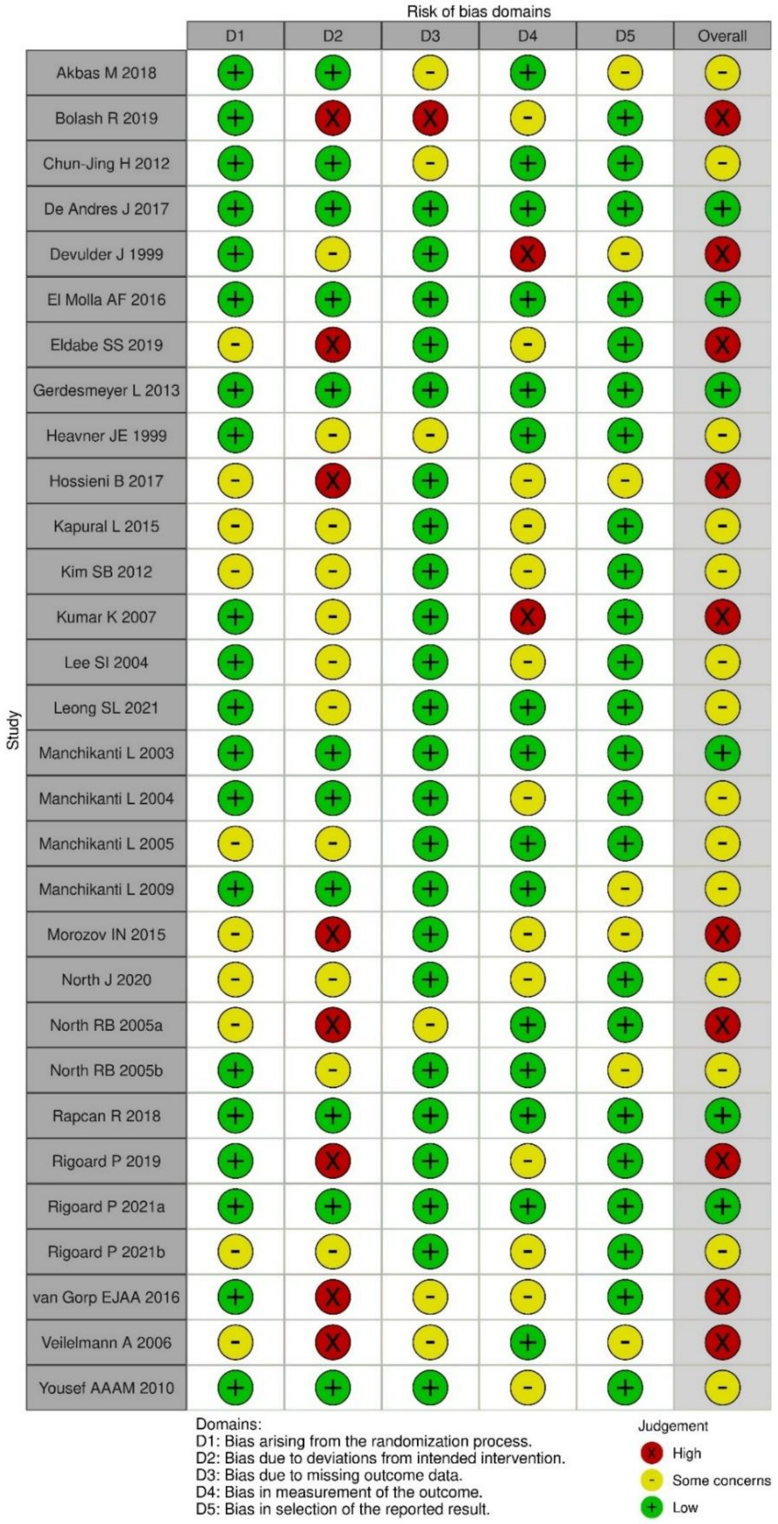

Figure S7. Risk of bias assessment for randomized trials (RoB 2 tool)

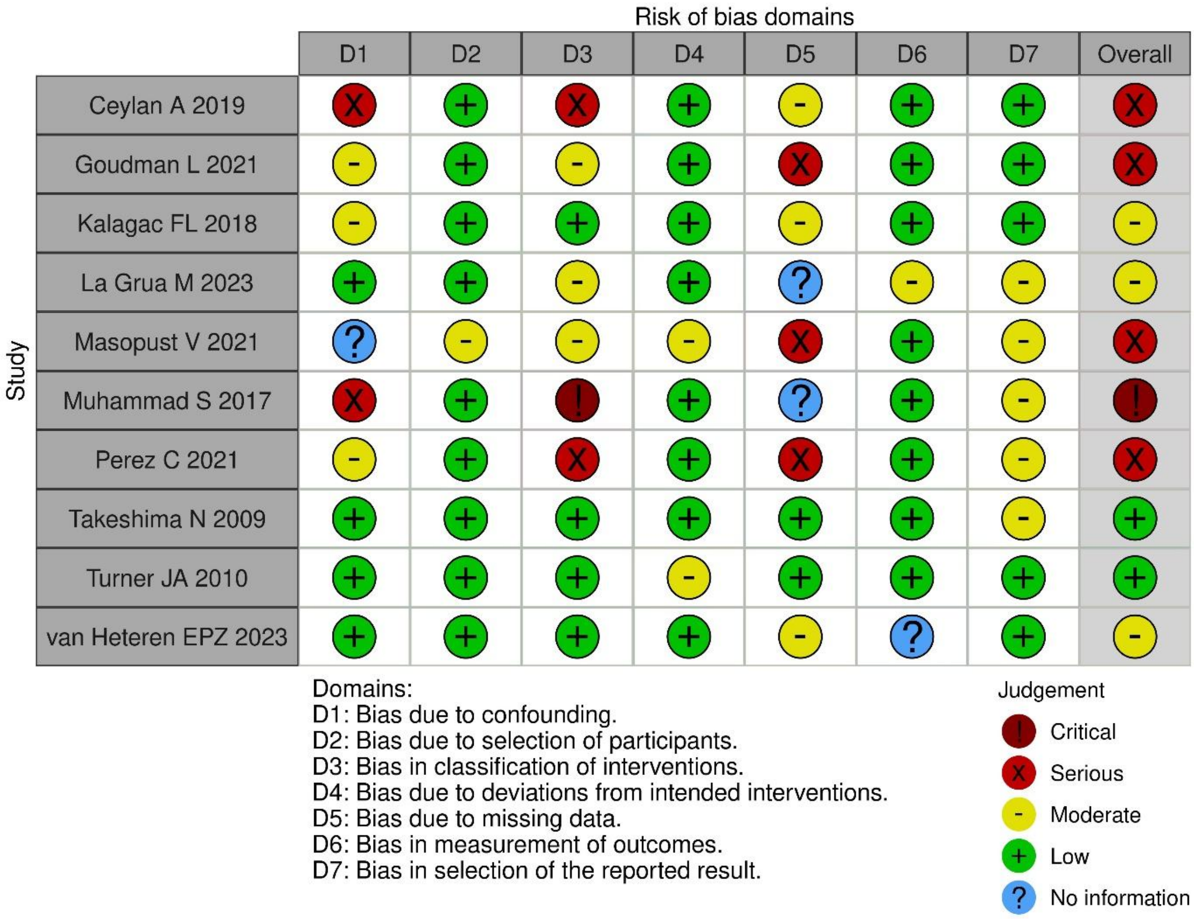

Figure S8. Risk of bias in non-randomized studies - of Interventions Tier 2 (ROBINS-I)

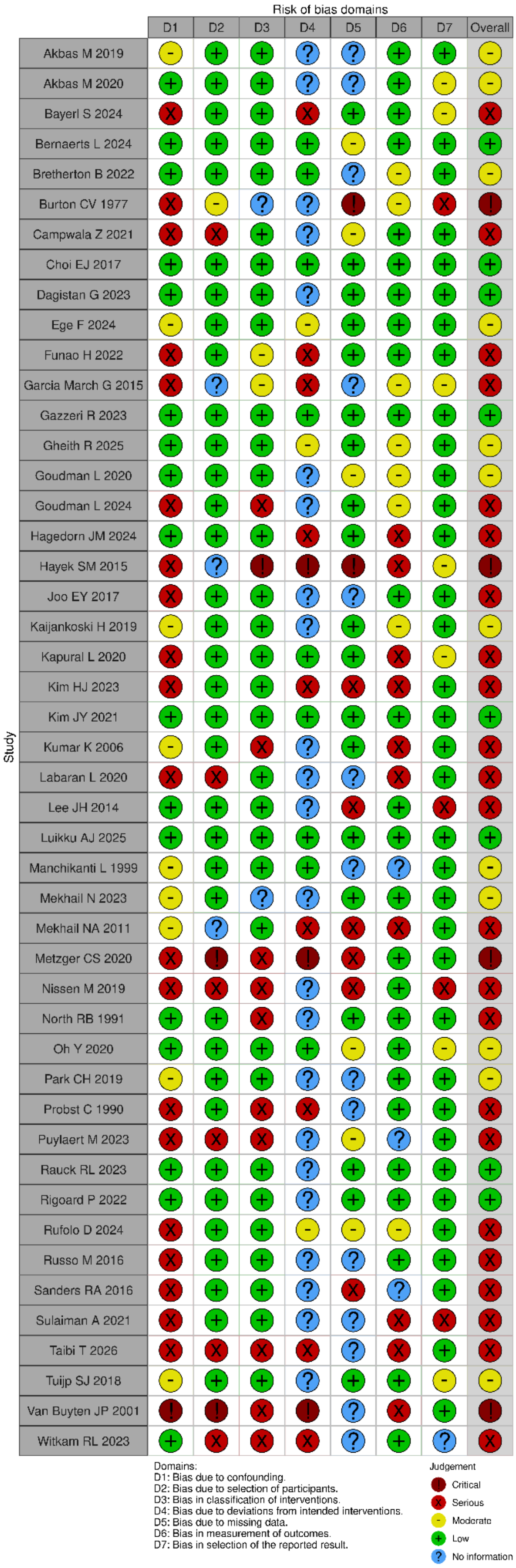

Figure S9. Risk of bias in retrospective studies – Tier 3 (ROBINS-I)
